# Ecological succession of fishes in a large Amazonian off river reservoir

**DOI:** 10.1038/s41598-025-07377-9

**Published:** 2025-07-02

**Authors:** Friedrich Wolfgang Keppeler, Marcos Augusto Mendes Rocha, Paulo Arthur de Abreu Trindade, Eurico Mesquita Noleto-Filho, José Amorim Reis-Filho, Jenny J. Morales-Parrado, Valter M. Azevedo-Santos, Ronaldo Angelini, Tommaso Giarrizzo

**Affiliations:** 1 Center for Aquatic Ecology and Amazon Fisheries, Federal University of Pará, Belém, Pará Brazil; 2 Civil Engineering Department, Federal University of Rio Grande do Norte (UFRN), Natal, Rio Grande do Norte Brazil; 3 Institute of Marine Sciences, Federal University of Ceará, Fortaleza, Brazil; 4 Geophysical Institute, University of Bergen, Bergen, Norway; 5 Graduate Studies Program in Ecology: Theory, Application and Values, Federal University of Bahia, Salvador, Brazil; 6 Postgraduate Program in Biodiversity, Ecology and Conservation, Federal University of Tocantins (UFT), Tocantins, Brazil

**Keywords:** Off-river reservoir, Ecological succession, Traits, Belo Monte, Fish assemblages, Amazon, Freshwater ecology, Ecology, Community ecology

## Abstract

Converting river flows into reservoirs is a major disturbance that triggers ecological succession, altering the functional and phylogenetic structure of aquatic communities. However, this process in off-river reservoirs remains understudied, despite its potential implications for biodiversity conservation. Here, we investigated fish assemblage colonization and establishment in a recently created off-river intermediate reservoir (IR) within the Belo Monte Hydroelectric Complex in the Amazon. Fish were sampled over seven years in the IR and compared to the main reservoir (MR) and a more pristine sector upstream (UP). Biodiversity in the IR increased over time, alongside shifts in species composition, traits, and phylogenetic structure. Dominance of large and benthic-rheophilic species rose, and the proportion of adults increased, indicating improved reproductive potential. These changes were likely influenced by environmental filtering, dispersal limitations, and resource scarcity during the initial years post-formation. Over time, the IR became more similar to the MR than the UP, reflecting species decay similarity and environmental resemblance between reservoirs. Dissimilarity between the IR and river sectors diminished as succession progressed. Our results suggest IR may serve as a refuge for endemic and rare rheophilic fish, supporting fish stocks for riverine communities and offering a conservation tool amid hydropower development and climate change. The true test of the IR’s value for local biodiversity will unfold as it ages, making consistent monitoring of the IR essential to assess its long-term ecological impact.

**Supplementary Information:**

The online version contains supplementary material available at 10.1038/s41598-025-07377-9.

## Introduction

As human interventions increasingly alter natural landscapes, understanding the processes that govern ecosystem recovery and community assembly has become critical for effective conservation and management. Ecological succession—the gradual and predictable shift in community composition over time—plays a vital role in informing restoration efforts and biodiversity management^1^. Understanding the nuances of succession is especially important in artificial ecosystems created by human activities to guide management towards desired ecosystem states^2^.

Ecological succession usually begins with increases in species richness, abundance, mean body size of individuals, and slow declines in rates of species gain and turnover^3^. Pioneer ‘opportunistic’ species, characterized by high dispersal, early maturity, rapid growth, and high reproductive effort, often dominate the early stages of succession. Over time, they are replaced by late-successional species that are better competitors, grow more slowly, mature later, have lower reproductive effort, and show greater resource specificity^4^. Ultimately, the succession process depends on local environmental factors, the evolutionary history, and the traits of the species in the regional species pool^5^.

Ecological succession studies have traditionally focused on terrestrial ecosystems, particularly plant communities^1^largely due to the challenges of observing aquatic organisms and the complexity of animal behavior and mobility. Recently, however, succession processes have garnered growing attention in aquatic ecosystems, including phytoplankton^6^, micro- and macroalgae^7^, aquatic macroinvertebrates^8^, and fish assemblages^9^.

Reservoirs formed by dam construction provide a compelling example of ecological succession in aquatic ecosystems, where shifts in community structure are driven by changes in habitat conditions. Following dam construction, rheophilic fish species that rely on long migrations to complete their life cycle tend to disappear due to the loss of lotic habitats, reduced reproductive stimuli, and disrupted river connectivity^10,11^. Species pre-adapted to lentic environments become increasingly dominant^12^. Additionally, unfavorable conditions in the benthic zone, such as low oxygen levels, can also reduce the diversity and abundance of benthic fish and macroinvertebrates^13^. These transformations typically result in reduced functional diversity and an increased risk of invasive species establishment, which can threaten local biodiversity and ecosystem function^14,15^.

In contrast, ecological succession in off-river reservoirs remains poorly understood. Unlike riverine reservoirs, off-river reservoirs are constructed parallel to a river rather than within its bed, receiving water through pumping or gravity^16^. They lack a pre-existing aquatic community, making them a unique case study for examining how fish species colonize and establish novel habitats. Fish colonization in these environments often depends on connectivity with surrounding water bodies, the physical structure of the reservoir, and the quality of available resources^17,18^. As a result, off-river reservoirs may inadvertently function as ecological traps, attracting species to suboptimal habitats that cannot support long-term population viability^19^.

**Fig. 1:**
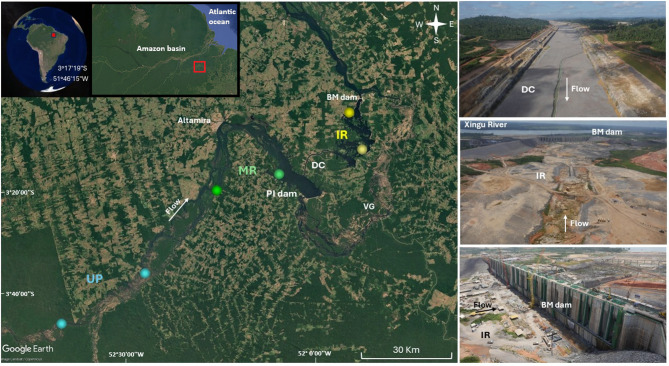
Sampling sites in the Middle Xingu River. Upstream (UP) sites are shown in blue, main reservoir (MR) sites in green, and intermediate reservoir (IR) sites in yellow. Panels on the right depict the construction process of the components forming the IR, including the diversion channel (DC; upper panel), IR (middle panel), and Belo Monte dam (BM dam; Lower panel). VG - Volta Grande, PI dam - Pimental dam. Red squares indicate the location of the study site. The satellite images were obtained from Google Earth 7.3.6 (https://earth.google.com/). Panels were combined and edited into a single figure using GIMP 3.0.2 (https://www.gimp.org/). Pictures credit: Norte Energia S. A.

Understanding the successional dynamics in off-river reservoirs is pivotal for assessing the ecological roles of these engineered ecosystems and for developing sustainable management strategies. Our study investigated the early stages of ecological succession in fish assemblages within an off-river reservoir at the Belo Monte hydroelectric complex, one of the largest in the world (Fig. 1). The Belo Monte complex has two dams, the Pimental dam, which intercepts the Xingu River, and the Belo Monte dam, which contains the main powerhouse (Fig. 1). The project was designed with run-of-the-river technology which causes little change to the original course of the river and is considered more sustainable^20^. To make the project viable, it was necessary to create an intermediate reservoir (IR), with 119 km², formed by 28 dikes and created on an area predominantly of dryland (Fig. S5). The IR was filled in 2016 and is connected to the main reservoir (created by the Pimental dam) by a diversion channel approximately 20 km long^21^. The water that enters the system is directed to the main power turbines of the project, located in the Belo Monte dam. Previous studies indicate that the operation of the project negatively affected fish abundance and diversity in the main reservoir (MR), the Volta Grande do Xingu (also known as the reduced flow section), and the sector downstream of the Belo Monte dam^22,23^. However, there is still no information on the process of colonization and establishment of ichthyofauna in the IR. Although fishing is common in the region, it is prohibited in the IR.

We explored changes across different biodiversity facets (taxonomic, functional, and phylogenetic) and examined the proportion of adults within and across species to evaluate their reproductive potential in the IR from 2016 to 2022. By taking a comprehensive approach to analyze colonization and assembly patterns in this artificial ecosystem, our study offers insights that could inform management practices for off-river reservoirs globally, supporting biodiversity conservation and ecosystem resilience in engineered landscapes. Five main hypotheses were tested: H1) There is a gradual increase in fish diversity in the IR after its creation attributed to ongoing enhancement of environmental conditions, ecosystem maturation, and continuum arrival of species from the river; H2) The fish assemblage of the IR (intermediate reservoir) represents a taxonomic subset of the main river’s assemblage, reflecting directional colonization from upstream sources and the role of environmental filtering in early community assembly; H3) The similarity between the IR and the riverine areas (UP and MR) increases over time as colonization progresses and environmental conditions in the IR converge with those in the river; H4) The early stages of colonization in the IR are dominated by small to medium-sized, pelagic, and generalist species, as these groups are more likely to persist under strong environmental filtering, limited niche availability, and structurally simplified habitats — conditions typical of newly formed aquatic systems; and H5) The proportion of adult individuals within the IR increases over time as environmental conditions stabilize and become more favorable for growth, survival, and recruitment.

## Results

### Diversity variation in the intermediate reservoir (IR)

**Fig. 2:**
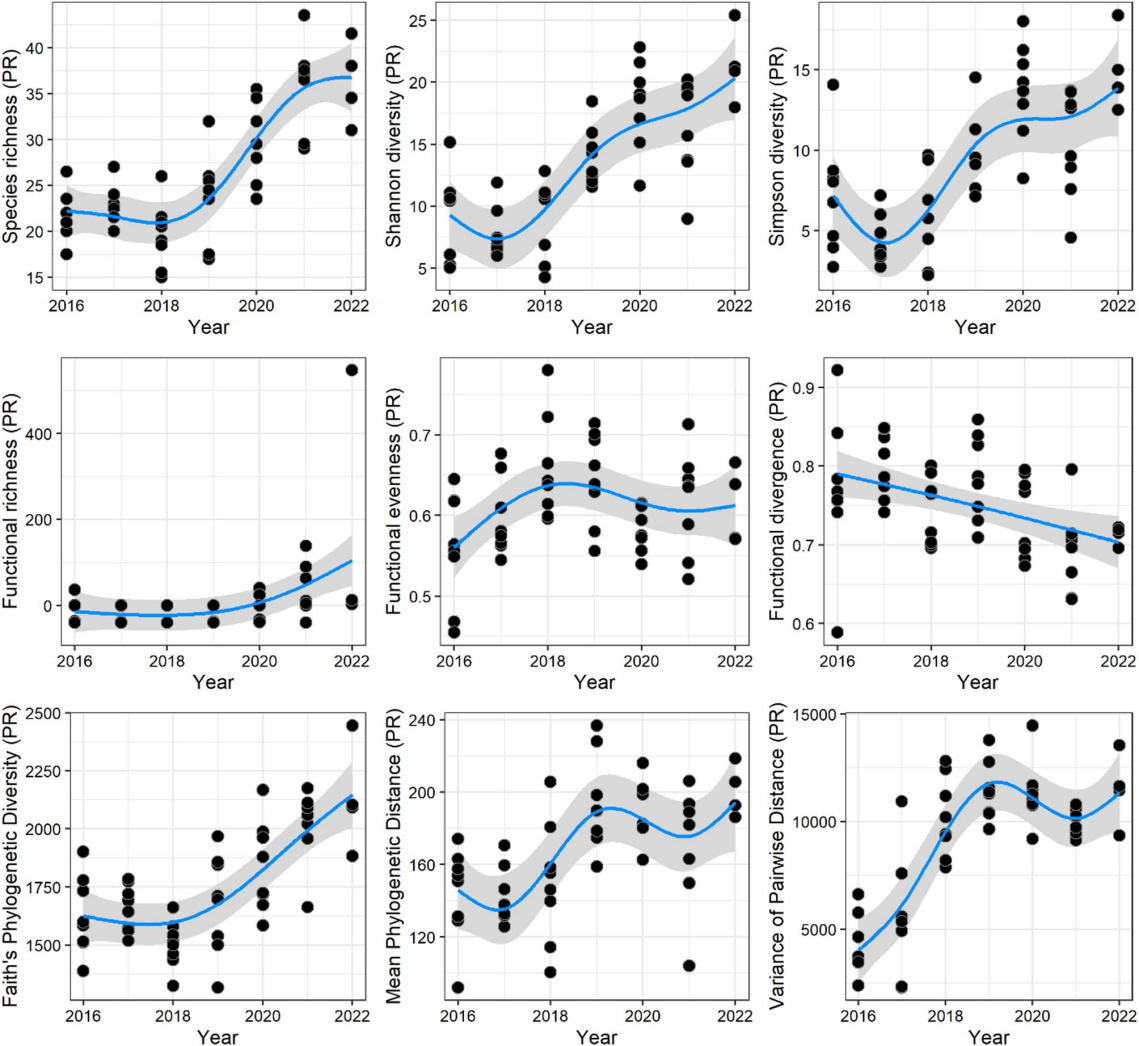
Variation of taxonomic (top panels), functional (middle panels) and phylogenetic (bottom panels) diversity metrics over the years of monitoring of the Intermediate Reservoir (IR) in the Xingu River (Brazil). Trend lines are shown in blue, with gray bars representing the standard errors. Points represent partial residuals (PR). Species richness, Shannon and Simpson diversities were calculated using Hill numbers (0, 1, and 2, respectively).

Significant changes in taxonomic, functional, and phylogenetic diversity were detected over the six years of monitoring in the IR. Species richness (F_(3.92)_ = 31.44, *P* < 0.001, Adjusted R² = 0.75), Shannon diversity (F_(3.88)_ = 21.25, *P* < 0.001, Adj.R² = 0.65) and Simpson diversity (F_(3.90)_ = 13.72, *P* < 0.001, Adj.R² = 0.54) remained practically constant in the first 3 years of monitoring but increased from 2018 onwards (Fig. 2).

Functional richness (F_(2.69)_ = 5.02, *P* = 0.005, Adj.R² = 0.27) remained constant until 2019 and then showed a sharp increase from 2020 onwards. Increases over time were also observed for functional evenness, but not for functional divergence. While functional evenness had an initial increase in the first three years (2016–2018) and then reached a plateau (F_(3.38)_ = 3.39, *P* = 0.025, Adj.R² = 0.17), the latter had a gradual decline over time (F_(1.19)_ = 9.79, *P* = 0.002, Adj.R² = 0.16) (Fig. 2).

All phylogenetic diversity metrics showed increasing patterns throughout monitoring. Faith diversity (F_(3.05)_ = 19.86, *P* < 0.001, Adj.R² = 0.67) remained stable until 2018 and then showed a constant increase until the end of monitoring. The mean pairwise distance (F_(3.98)_ = 7.69, *P* < 0.001, Adj.R² = 0.43) and the variance of the pairwise distance (F_(3.98)_ = 31.40, *P* < 0.001, Adj.R² = 0.75) showed similar patterns, with an increase in the metrics until 2019 and then stagnation until the end of monitoring (Fig. 2).

### IR as a subset of the upstream (UP) and main reservoir (MR) communities

A total of 133 species were found in the IR (Table S1). Of these, 15 species were not found in either the MR, which had a total of 153 species, or the UP, with 147 species (Fig. S1). The IR had a total of 102 species (77% of the total richness of the IR) shared with both the MR and the UP. More species were shared between the IR and the MR (114 species) than between the IR and the UP (106 species).

Most of the difference in species composition between the IR and the river (MR and UP) was explained by species replacement rather than species loss (i.e., the difference in species richness). This pattern was consistent across all the different facets of biodiversity analyzed (i.e., taxonomic, functional, and phylogenetic) (Fig. S2).

### Dissimilarity of the IR assemblage with the assemblages of the UP and MR sectors

**Fig. 3:**
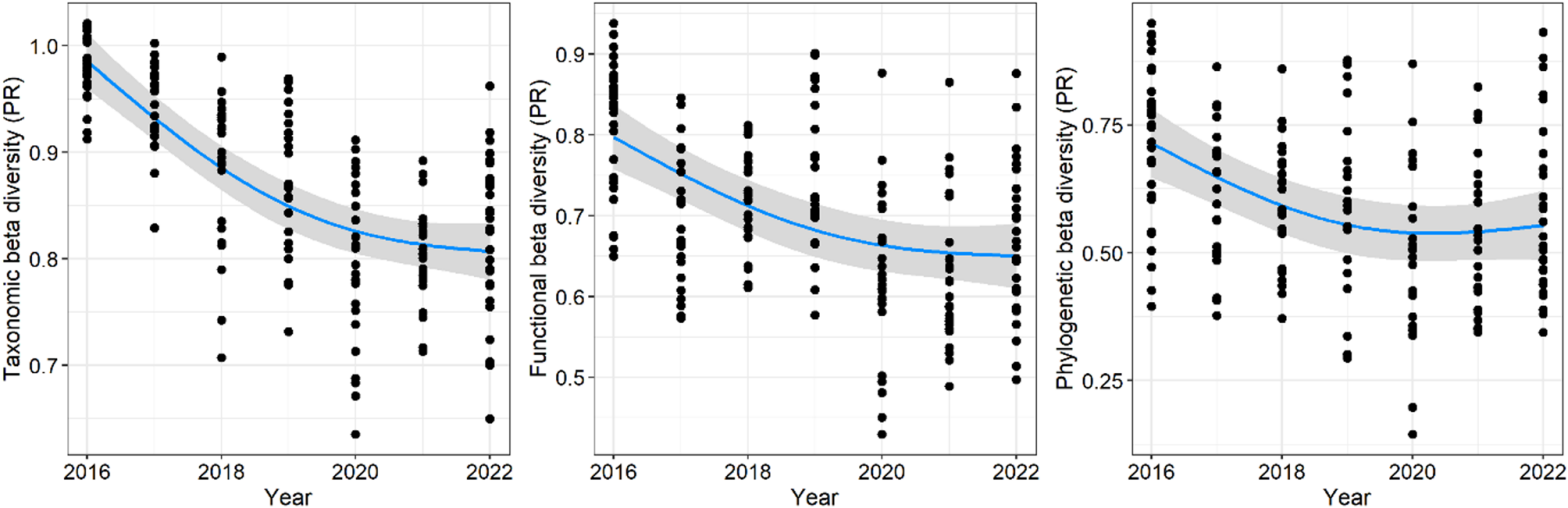
Temporal variation of taxonomic, functional and phylogenetic beta diversity between fish assemblages in the Intermediate Reservoir (IR) and assemblages sampled in the river. Trend lines are shown in blue, with gray bars representing the standard errors. Points represent partial residuals (PR) to remove the effect of confounding variables.

A decline in beta diversity was observed between the IR and the sectors sampled in the river (UP and MR) over the years (Fig. 3). This pattern was consistent in both taxonomic (F_(1.99)_ = 101.58, *P* < 0.001, Adj.R² = 0.55), functional (F_(1.95)_ = 33.58, *P* < 0.001, Adj.R² = 0.32), and phylogenetic (F_(1.98)_ = 15.04, *p* < 0.001, Adj.R² = 0.28) beta diversity. Beta diversity indices were lower between the IR and the MR than between the IR and the UP sector (F_(3)_ = 2.73, *P* = 0.01).

### Variation in the functional structure of the IR assemblage

**Fig. 4:**
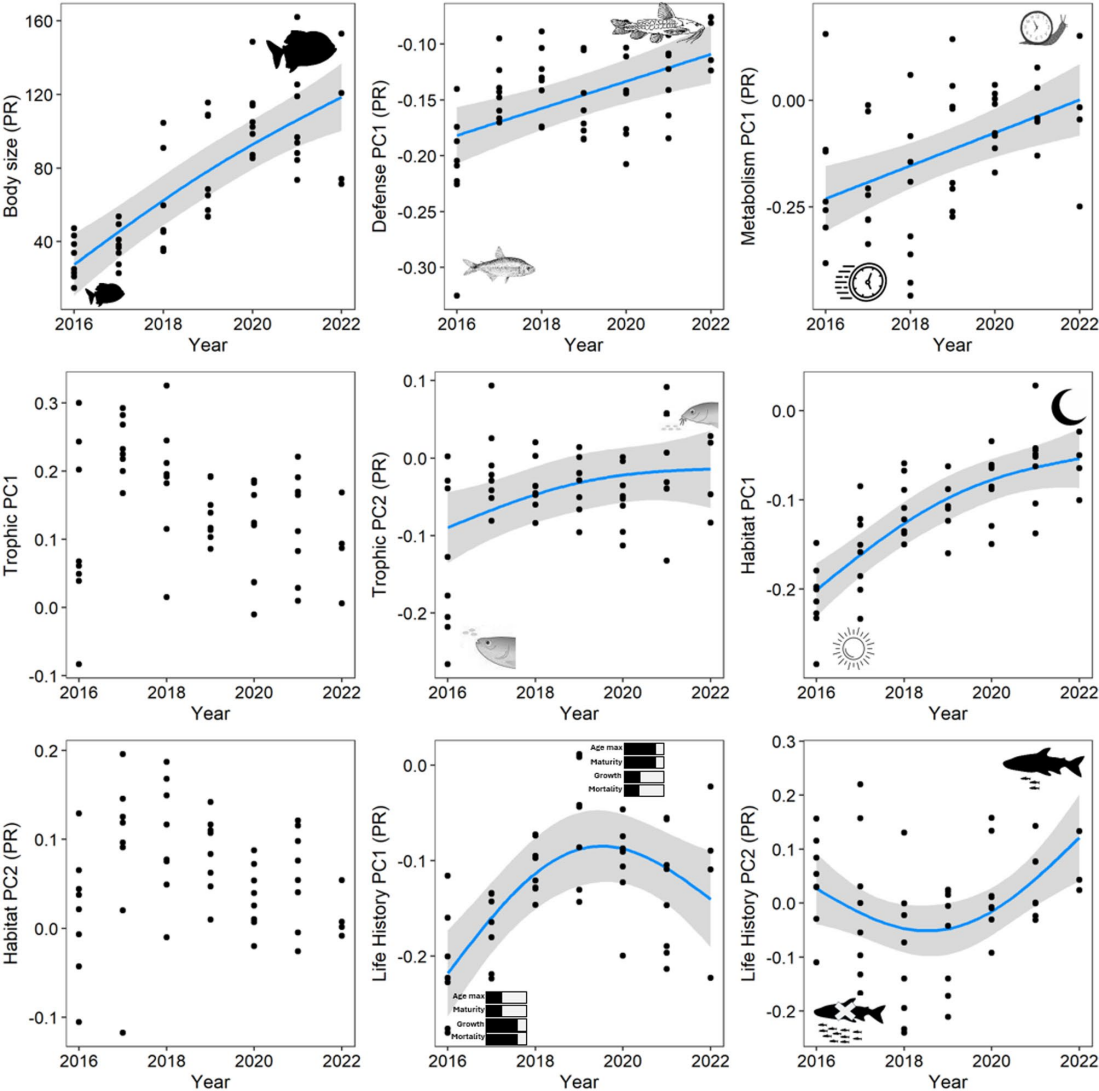
Temporal variation of the functional structure of fish assemblages in the intermediate reservoir (RI). Trend lines are shown in blue, with gray bars representing the standard errors, unless no significant temporal variation was detected. Except for body size, all the other traits are principal axes generated to reduce data dimensionality (see Table S2). Small icons, symbols, and visual elements within the subplots indicate the functional traits assessed.

Body size increased more than 3 times from 2016 (~ 38 mm) to 2022 (~ 120 mm) (F_(1.65)_ = 48.32, *P* < 0.001, Adj.R² = 0.61) (Fig. 4). The main axis of the defense dimension was positively associated with the sampling years (F_(1.00)_ = 16.13, *P* < 0.001, Adj.R² = 0.27), indicating that species with low aggressiveness, low speed, large number of spines and armor become gradually more common. The main axis of metabolism was positively associated with the sampling years (F_(1.00)_ = 13.38, *P* < 0.001, Adj.R² = 0.22), suggesting an increasing dominance of sedentary species with lower oxygen tolerance over time. Notably, most sedentary species were loricariids with low metabolic rates, yet they have low tolerance to oxygen depletion, as they are adapted to fast-flowing, well-oxygenated waters in rapids.

The second main axis of the trophic dimension (F_(1.76)_ = 5.71, *P* = 0.024, Adj.R² = 0.27) and the first dimension of habitat (F_(1.95)_ = 43.18, *P* < 0.001, Adj.R² = 0.66) were positively related with sampling year as species with inferior mouths and nocturnal habitats become more common with time (Fig. 4). Both the first (F_(1.99)_ = 15.17, *P* < 0.001, Adj.R² = 0.59) and the second axis (F_(1.99)_ = 5.56, *P* = 0.005, Adj.R² = 0.19) of the life history dimension had significant associations with the sampling years. In both cases, the trends were positive, although not linear: PC1 values peaked in 2019–2020 and then slightly declined until 2022, whereas the PC2 values slightly declined until 2019 and then gradually increased until the end of the monitoring period. These results show that fishes with parental care, viviparity, low fecundity, late senescence, late maturity, slow growth, and low mortality became more dominant over time.

### Variation in the proportion of adults in the IR

**Fig. 5:**
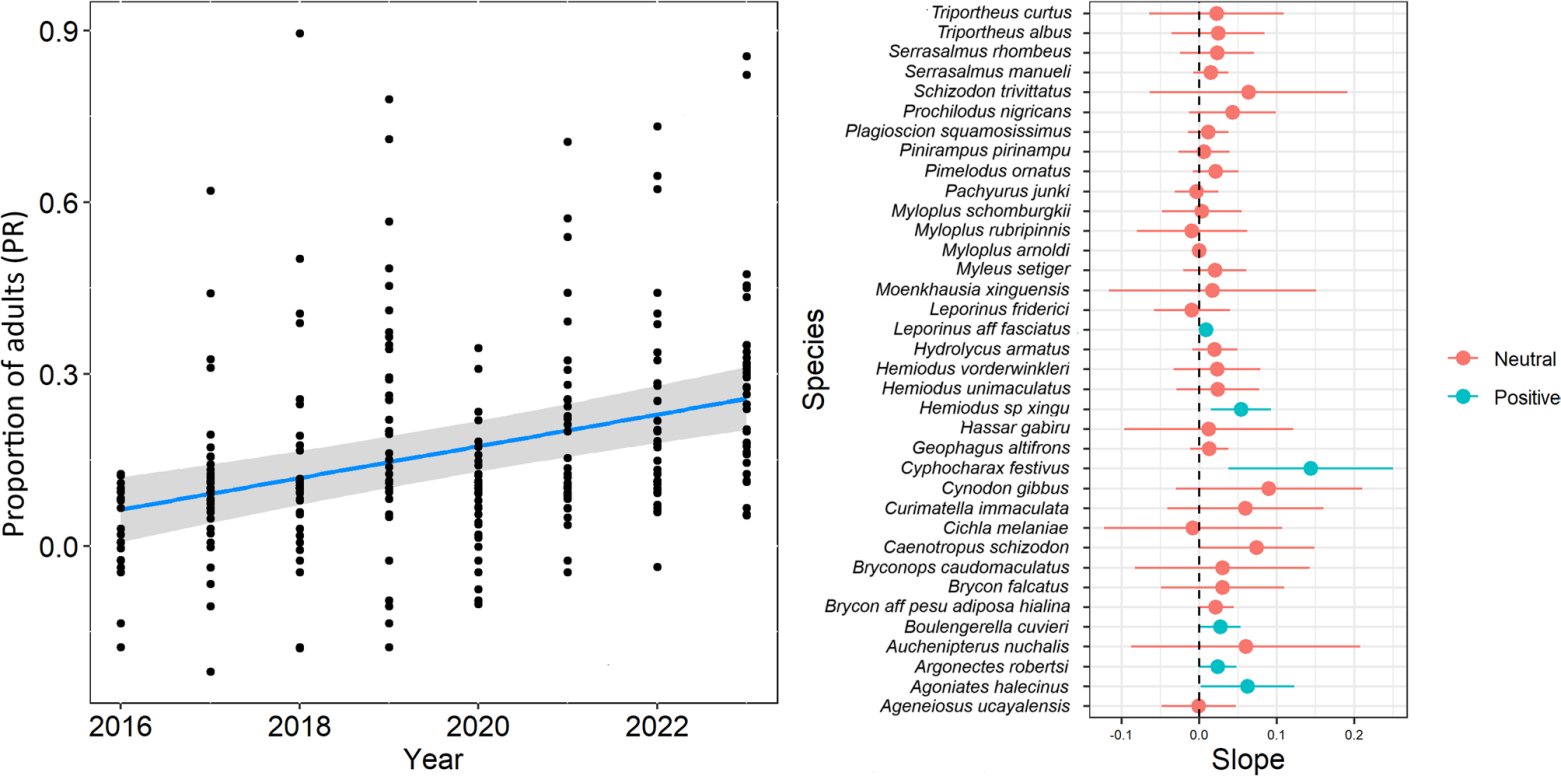
On the left, the relationship between sampling year and proportion of adults is presented. Points are partial residuals (PR). Trend lines are shown in blue, with gray bars representing the standard errors. On the right, a forest plot displays the slope coefficients from linear regressions analyzing the relationship between proportion of adults and years for each fish species. Significant relationships are those where the confidence interval (horizontal line) does not include zero (dashed line). Relationships for each species can be seen in Fig. S3.

A positive relationship was observed between the sampling year and the proportion of adults (t_(237)_ = -5.60, *P* < 0.001, marginal R^2^ = 0.08, conditional R^2^ = 0.36). On average, the proportion of adults increased from ~ 6% in 2016 to ~ 26% in 2022 (Fig. 5). Six species had consistent increases in the proportion of adults while the remaining had neutral relationships (Fig. 5; Fig. S3).

## Discussion

The results indicate that the IR created in 2016 already harbors a significant portion of the fish diversity found in the Middle Xingu River. Profound changes in diversity and species composition were observed following the creation of this off-river reservoir. Although not necessarily linear, increases in fish diversity were observed, supporting H1. The IR fish assemblage was not a subset of the main river’s (H2), as it exhibited high diversity and differences in species composition were mainly due to species replacement. As expected in H3, the ichthyofauna structure gradually resembled that of the river over time. The ichthyofauna showed a gradual increase in dominance of large-bodied and bottom-associated species, confirming H4. Finally, a temporal increase in the proportion of adult fish was observed, supporting H5 and suggesting an increase in reproductive potential in the IR. These results indicate that there is a continuous process of ecological succession in the IR. The biggest question remains about the continuity of this process and whether this artificial environment can become an important area for biodiversity conservation as has occurred in other artificial water bodies worldwide^24,25^.

The Belo Monte hydroelectric complex has always raised major concerns due to its size and expected impact on the ichthyofauna and riverine communities that depend on this resource, especially in the Volta Grande (VG), a sector with reduced water flow located downstream of the Pimental dam^26,27^. Recent studies indicate that there have been reductions in the abundance and diversity of fish and fisheries in the region^22,23,28^. Here, we showed that the IR is an exception, exhibiting the opposite pattern in its first years after creation. This outcome is unexpected, as the project did not anticipate an increasing pattern^29^.

The low diversity of fish observed in the first years of monitoring suggests a strong environmental filtering process associated with inhospitable conditions after the IR was filled. Among these, low water quality (e.g., high turbidity, low dissolved oxygen levels and pH values; Table S3, Fig. S4) stands out due to the high volume of suspended material originating from a still unconsolidated reservoir bed. Species with low tolerance to dissolved oxygen and those associated with the bottom, a critical region that generally presents the most adverse conditions^10,13^, were poorly represented in the first samplings. The early dominance of species with opportunistic strategies, such as fast growth and high reproductive effort, suggests the presence of stressed environments with low predictability^30^. Furthermore, low habitat complexity, and availability of prey and basal resources may have limited the initial establishment of the ichthyofauna. While no direct evidence (e.g., massive mortality events) was detected, the system likely functioned as an ecological trap^31^ at this stage for some species, exposing them to elevated mortality risks and sublethal impacts, such as reduced condition factor and impaired reproductive activity under harsh environmental conditions.

As the system matured and environmental conditions consolidated, there was an increase in fish diversity. This pattern was not restricted to the number of species but also involved more profound changes in the functional and phylogenetic structure of the assemblage. Colonization by different functional groups with distinct evolutionary histories suggests that the connection through the diversion channel is efficient and even allows access by sedentary species, such as loricariids, a diverse family of armor catfishes^26,32^. Furthermore, there appears to have been an increase in the availability of vacant niches, with species occupying different trophic levels and habitats that were non-existent (e.g., macrophyte banks) or inaccessible due to harsh conditions.

The lower diversity in the initial years after the creation of the IR might be attributed to the early colonization by a few closely related dispersers with similar traits^33^. During these early years, we noted the prevalence of small characids, such as *Astyanax bimaculatus* and *Ctenobrycon spilurus* (see Table S1). Interestingly, small species often have higher energy costs per unit of mass for movement compared to larger species, which typically exhibit better dispersal capabilities and larger home ranges^34^. Small characids are active swimmers with pelagic behavior, which, along with their high abundances^35^, may enhance their capacity to colonize new areas^36^. Conversely, a diverse array of small to medium-sized rheophilic species from the Xingu River, which exhibit sedentary behavior and equilibrium life history strategies, were only observed in the later years of our study (e.g., *Hypostomus plecostomus, Aphanotorulus emarginatus*). These species, characterized by lower fecundity and parental care^26,37^likely have poorer dispersal capabilities. Nonetheless, dispersal limitation alone may not fully explain the patterns observed. Many medium to large pelagic species with high dispersal potential (e.g., dogtooth characins, halftooths) were less dominant in the IR during the initial years, suggesting that other factors may also be influencing the observed successional dynamics. One likely explanation for the rapid colonization of the IR by small species is that some small streams, intercepted by the dam, provided a crucial early source of fish diversity.

The increases in taxonomic and phylogenetic diversity were overall more intense than those observed in functional diversity. Functional divergence declined whereas functional richness and evenness increased at the end and beginning of the monitoring period, respectively. Together, these results suggest that the functional space of the assemblage expanded with species presenting similar abundances and occupying complementary areas of the functional space^38^. This may reflect the availability of new opportunities in the ecosystem, including habitats and resources. The functional expansion was less intense than the increase in taxonomic diversity, which is expected given the high levels of trait redundancy in Neotropical ichthyofauna^39^. Conversely, the fast increment in phylogenetic diversity metrics suggests that the diversification in the IR involved species from multiple clades. Not only that, but the average phylogenetic distance among species increased with time, but this increment was not homogeneous. In other words, the occurrence of unique lineages of fish (e.g., freshwater stingray) disproportionally increased the phylogenetic space whereas, at the same time, several new species that colonized the IR were from related species (e.g., anostomids).

The ecological succession of the IR involved all the five niche dimensions analyzed here (metabolism, defense, habitat, trophic, and life history) via functional traits. Fishes with large body sizes, late maturation and senescence, and slower growth become increasingly more common in the IR. These are characteristics of species with equilibrium and periodic strategies^40^, many of which present lateral and longitudinal migration patterns, such as pacus, giant catfishes, and peacock bass^41^. This group of fish is among the top species composing the fish landing of the riverine population in Amazon rivers and, therefore, has a pivotal role in food security and the provision of protein^42^. In addition, more sedentary fishes with inferior mouths, parental care, low fecundity, heavy armor, and low oxygen tolerance occurred more often and at higher abundance after the first years of the monitoring program. These are typical characteristics of rheophilic fishes, which inhabit the bottom of the reservoir and the derived channel, the latter exhibiting features reminiscent of rapids, such as swift currents during high water and a rocky substrate. Rheophilic fishes are among the most threatened groups of fish in the region due to the reduction of water flow to the VG^26,37^. In this sense, these artificial environments could become important elements for the conservation of rheophilic species in the region.

During the study period, the fish assemblages of the IR became more similar to those present in the MR, as an already altered environment, and even to those of the UP sector, which retains more natural environmental conditions. While the MR and UP also changed over time, their community shifts were less pronounced and less directional than the succession-driven changes in IR^43^ (Fig. S7 and S8). Thus, the increasing similarity between the IR and the UP/MR can be attributed directly to the succession process in the IR. Additionally, the degree of similarity was directly related to the distance of the sectors, where the IR was more similar to the MR than the UP. The decay of assemblage similarity distance is a pattern widely observed in nature and can be explained purely by neutral processes, such as random dispersal, dispersal limitation, and ecological drift^44^. However, the detected pattern may also be associated with the niche of the species and their response to environmental conditions, since there is greater environmental similarity between the IR and MR, given that both sectors are reservoirs with greater depth and lower water velocity.

The dominance of species replacement over species loss and gain was unexpected. While 15 rare species were found exclusively in the IR, the differences in assemblage structure between IR and UP, as well as between IR and MR, appear to be primarily driven by variations in species abundance rather than turnover in species occurrence. For example, *Serrasalmus rhombeus*, the most abundant species in the IR (with a relative abundance of 16%), ranked outside the top 10 in both the UP (2%) and MR (1%) sectors. Distinct environmental conditions within the IR, such as increased unpredictability in water fluctuation and longer water residency times, likely favor species that are less dominant in the main river.

The increase in adult proportion in the IR indicates that the ecosystem is maturing^45^. A higher proportion of adults suggests greater reproductive potential, lower mortality rates, and, consequently, more stable populations^45,46^. However, it may also indicate aging populations that struggle to recruit new cohorts. One possibility is that lower juvenile dominance reflects higher predation rates as larger fishes become more dominant in the system. Conversely, environmental conditions in the IR are improving, which is likely to benefit juvenile fish and support recruitment. Ichthyoplankton data show a consistent presence of fish larvae and eggs in the IR, with fish larvae peaking in occurrence in 2021^43^. Even if recruitment conditions in the IR are suboptimal, adults—having larger home ranges than juveniles^34^ —may disperse to nursery areas in the river. Future studies on juvenile fish dynamics, recruitment, and adult fish movements could clarify the patterns observed here.

Reservoirs are generally seen as inhospitable environments with poor biodiversity. Although there is an initial period of trophic ascension in traditional reservoirs with increases in fish richness and abundance fueled by the decomposition of flooded organic matter^10^, there is a subsequent decline and subsequent stabilization of these metrics in a period ranging from 15 to 40 years after damming^11^. In the case of the IR, the diversification process, which has already lasted 7 years, is more associated with the colonization process that has been driven by species from the main river channel and small streams that have been intercepted by the dam. This process should slow down as the diversity of the IR equalizes with that of the MR. Noteworthy, the IR fish assemblage shows signs of sustainability, with a gradual increase in the number of adult fish and the occurrence of spawning^21^. Although there is still no evidence of fish spillover to adjacent regions of the river, it is possible that the IR will become a source of biomass and diversity in the region. This expectation is given that the IR has a large area and is in a private area belonging to the project concessionaire (Norte Energia), which prohibits fishing activities and protects riparian forests. Studies show that protected freshwater areas can supply fish to adjacent areas^47,48^.

The potential of the IR for preserving fish stocks is attractive, but expectations must be adjusted. First, the IR should benefit species that are adapted to more lentic environments and that do not need to undertake long-distance migrations to complete their life cycle. Among these, species of commercial and food security interest can be highlighted, such as peacock bass, silver croaker, and piranhas. Second, we still know little about the ecological succession of off-river reservoirs. However, it is anticipated that the Middle Xingu River will experience a decline in species diversity as the full impacts of the Belo Monte dam unfold. This reduction could negatively affect riverine communities that rely on the river’s fishing resources for their livelihoods^22^. Third, the increase in diversity observed in the IR may functionally resemble the trophic upsurge seen in other river reservoirs^11^ and could begin to decline in the coming decades as the reservoir ages^49^. In this sense, it would be prudent to take measures that maximize the productivity and diversity of the IR. For example, artificial habitats could be installed to increase local structural complexity and provide areas for fish spawning and foraging^3^. In this context, monitoring of aquatic fauna would be essential to assess the efficiency of the measures taken and potentially propose adjustments to the management and conservation plan.

Our study showed a rapid, directional colonization and establishment of fish in a large off-river reservoir in the Amazon basin. We demonstrated the presence of a strong initial environmental filter, which was exacerbated by limited resources and dispersal constraints, restricting the presence of several fish lineages and functional groups. This includes sedentary, benthic species sensitive to oxygen depletion and large species with slower growth and senescence rates. These are novel results that clarify our understanding of ecological succession processes in aquatic environments. We also highlighted the potential of this artificial ecosystem for fish conservation and supporting riverine communities.

## Methods

### Study area

The Belo Monte complex is the third largest in the world and is located on the Xingu River, a large tributary of the Amazon River^22^. The Xingu River has transparent and greenish waters with few dissolved solids and sediments^50^. The river has four distinct hydrological periods: rising water (December/February), high water (March/May), falling water (June/August), and low water (September/November)^51^. The region has a tropical climate with a high average temperature (around 27.9 °C), with few fluctuations during the year. Its waters harbor around 450 species of fish, 63 of which are endemic, and 160 are rheophilic species present mainly in the VG^37^.

### Fish sampling and gonadal analysis

Fish were collected from 2016 to 2022 at six sampling sites: two in the IR and four in two reference sectors—the MR and an upstream area (UP) with environmental characteristics similar to the pre-dam phase (Fig. 1). Collections were carried out quarterly, encompassing the four main periods of the hydrological cycle of the Xingu River. Fish were collected with three sets of gillnets of different mesh sizes (2, 4, 7, 10, 12, 15, and 18 mm), 20 m long and 2 m high, in open waters close to the shoreline, remaining for 17 h, between 4 pm and 9 am. After collection, fish were anesthetized with eugenol, identified to the species level, and reference specimens were fixed in 10% formalin and subsequently conditioned in 70% alcohol. Subsamples of the collected fish were dissected to assess the stage of gonadal maturation. Fishes were classified as juvenile and adult according to the macroscopic approach proposed by Vazzoler^52^..

### Functional traits

Twenty-eight morphological measurements (Table S4) were taken from the collected fish species to calculate 26 functional traits related to feeding and habitat use (Table S5). Measurements were made on three adult individuals per species. We restricted measurements to adults to minimize ontogenetic variation, as our goal was to examine interspecific differences and community structure. The morphological measurements were made with a digital caliper (mm). Fish were also classified into 6 functional groups linked to their type of metabolism and defense (Table S6). We also obtained 7 functional traits linked to life history using the FishLife R package (Table S6)^53^.

### Phylogeny

The phylogenetic proximity of the species was estimated using the supertree available in the fishtree R package^54^. This phylogenetic tree is composed of more than 11,000 fish calibrated using fossil records. The phylogenetic distance was resolved at the genus level (Fig. S6) as many species collected are not yet present in the tree. Previous research indicates that using genus-level phylogenies is effective for examining phylogenetic community structure^55^.

### Diversity metrics

Taxonomic, functional, and phylogenetic diversity were calculated for each site in each sampling event. Taxonomic diversity was assessed using three Hill numbers: q = 0, which is equivalent to richness (equal weight to rare and abundant species); q = 1, which is equivalent to the Shannon diversity (considers all species proportionally); and q = 2, which is equivalent to the Simpson diversity (more weight to abundant species)^56^. Functional diversity was calculated using three main indexes: (1) richness (volume of functional space occupied by the assemblage); (2) divergence (divergence of species abundance within the functional space); and (3) evenness (uniformity of species abundance in the functional space)^57^. Functional diversity metrics were constructed using 38 functional traits: length, mouth position, and gill rakers (Table S4), along with those listed in Table S5 and S6. Phylogenetic diversity was estimated using three main metrics: (1) Faith’s index (sum of the lengths of all branches of the phylogenetic tree connecting all species in the assemblage); (2) mean phylogenetic distance (average distance between pairs of species in the phylogenetic tree); and (3) pairwise distance variance (variation in distance between pairs of species in the phylogenetic tree)^58^. We selected these metrics because they are largely independent and represent key aspects of taxonomic^56^, functional^57^, and phylogenetic diversity^58^.

### Data analysis

We used Hierarchical Generalized Additive Models (HGAM)^59^ to explore the temporal variation for taxonomic, functional, and phylogenetic diversity indices in the IR (H1). Diversity indices were the response variables, with sampling year as the predictor. We included sampling period (e.g., high, falling water) and site as random effects in the model to control confounding effects.

To test whether the fish assemblage in the reservoir is a subset of those in the upstream and reservoir sectors (H2) and whether these assemblages have become more similar over time (H3), a Venn diagram was created, and beta diversity was calculated between the sampled and reference sites (MR and UP) for each study year. For the Veen diagram, we pooled all species sampled in each sector (IR, MR, and UP) during the entire study period. Beta diversity was partitioned into two main components: species gain/loss (differences in richness) and species replacement to further test H1. If the IR is indeed a subset of the main river, then the species gain/loss component should be more significant in explaining species composition differences than species replacement. The calculation of beta diversity and its partition followed the methodology proposed independently by Podani & Schmera^60^ and expanded by Cardoso et al.^61^ for functional and phylogenetic diversity. The variation in beta diversity over the years was modeled using HGAM. Beta diversity was the response variable, sampling year was the predictor, and period and site were the random effects.

Community-weighted means (CWM) were used to investigate temporal changes in functional traits in the IR (H4). To simplify the variety of traits used in the analysis, we group the data into five main niche dimensions (trophic, habitat, defense, metabolism, and life history) and then principal coordinates analysis (PCoA - Gower distance) or principal component analysis (PCA, only for traits linked to life history) was used to reduce the complexity of each niche dimension into one or two main axes^62^. The axes generated by the ordinations and their correlations with the traits are presented in Table S2. Body size (Standard Length - SL) was also included independently in the analysis since this trait is related to multiple niche dimensions. We used HGAM with year as predictor and hydrological periods and sites as random effects to explore the temporal variation of each averaged (CWM) trait dimension.

Finally, a linear mixed-effects model (LMM) was used to investigate changes in the proportion of adults (dependent variable) throughout the monitoring years (independent variable) (H5). Fish species were included as a random effect (intercept) in the model to account for the lack of independence among samples due to variations in reproductive biology. Only species that occurred in at least six of the seven years were used. We also conducted simple linear regressions for each species to further investigate which species changed the proportion of adults over the years.

The analyses were conducted using seven R core team packages (mgcv^63^, BAT^64^, vegan^65^, FD^66^, picante^67^, VennDiagram^68, ^and lme4^69,70^. We chose HGAM to model variations in the metrics of interest due to its flexibility in capturing non-linear patterns. The temporal dynamics depicted in the figures were obtained using partial residuals (i.e., conditional plots) to account for confounding factors^71^ specifically spatial effects and hydrological periods in the HGAM, and species-specific variations in the proportion of adults in the LMM. Partial residuals are a commonly used diagnostic and visualization tool in multiple regression, calculated by subtracting the effects of all other predictors from the observed response variable, leaving only the residuals associated with a specific predictor of interest^71^. This approach also aids in evaluating variability, identifying outliers, and detecting potential deviations from model assumptions^71^.

All methods were carried out in accordance with relevant guidelines and regulations.

## Electronic supplementary material

Below is the link to the electronic supplementary material.

Supplementary Material 1

## Data Availability

The datasets used and/or analyzed during the current study available from the corresponding author on reasonable request.
