# Supplementary material for: Ecological succession of fishes in a large Amazonian off river reservoir
Source: Sci Rep. 2025 Jul 2;15:22616. doi: 10.1038/s41598-025-07377-9 (PMC12216564; doi:10.1038/s41598-025-07377-9)
Supplement: Supplementary file 1 — Supplementary Material 1 [file 41598_2025_7377_MOESM1_ESM.docx]

**Supplementary Materials**

**Table S1.** Abundance of fish species of the intermediate reservoir (IR) in each study year.

| **Order** | **Family** | **Species** | **2016** | **2017** | **2018** | **2019** | **2020** | **2021** | **2022** |
| --- | --- | --- | --- | --- | --- | --- | --- | --- | --- |
| Myliobatiformes | Potamotrygonidae | *Potamotrygon motoro* | **-** | **-** | **-** | **-** | 1 | **-** | **-** |
| Myliobatiformes | Potamotrygonidae | *Potamotrygon orbignyi* | **-** | **-** | **-** | **-** | **-** | **-** | 1 |
| Gymnotiformes | Gymnotidae | *Gymnotus carapo* | 1 | **-** | **-** | **-** | **-** | 1 | **-** |
| Gymnotiformes | Rhamphichthyidae | *Rhamphichthys marmoratus* | **-** | 1 | **-** | **-** | **-** | **-** | **-** |
| Gymnotiformes | Sternopygidae | *Eigenmannia trilineata* | 1 | **-** | **-** | **-** | **-** | 1 | 1 |
| Characiformes | Erythrinidae | *Hoplias aimara* | 1 | 1 | 1 | **-** | **-** | **-** | **-** |
| Characiformes | Erythrinidae | *Hoplias malabaricus* | 26 | 6 | **-** | **-** | **-** | 1 | **-** |
| Characiformes | Cynodontidae | *Cynodon gibbus* | **-** | 7 | 20 | 5 | 12 | 5 | 5 |
| Characiformes | Cynodontidae | *Hydrolycus armatus* | **-** | 18 | 39 | 35 | 57 | 82 | 43 |
| Characiformes | Cynodontidae | *Hydrolycus tatauaia* | **-** | 2 | 6 | 6 | **-** | 6 | 7 |
| Characiformes | Cynodontidae | *Rhaphiodon vulpinus* | **-** | 1 | **-** | 1 | **-** | **-** | 1 |
| Characiformes | Serrasalmidae | *Acnodon normani* | **-** | **-** | **-** | 7 | 16 | 16 | 11 |
| Characiformes | Serrasalmidae | *Serrasalmus manueli* | 2 | 1 | 9 | 2 | 11 | 37 | 16 |
| Characiformes | Serrasalmidae | *Serrasalmus rhombeus* | 709 | 873 | 415 | 209 | 155 | 100 | 42 |
| Characiformes | Serrasalmidae | *Tometes ancylorhynchus* | 7 | 3 | 3 | **-** | 2 | 7 | **-** |
| Characiformes | Serrasalmidae | *Tometes kranponhah* | **-** | 3 | **-** | **-** | 1 | 9 | **-** |
| Characiformes | Serrasalmidae | *Metynnis luna* | 1 | **-** | **-** | **-** | **-** | **-** | **-** |
| Characiformes | Serrasalmidae | *Myleus setiger* | 1 | 4 | 18 | 23 | 26 | 27 | 8 |
| Characiformes | Serrasalmidae | *Myloplus arnoldi* | 1 | 1 | **-** | 1 | 2 | 1 | 1 |
| Characiformes | Serrasalmidae | *Myloplus asterias* | 4 | 2 | 1 | 1 | **-** | **-** | **-** |
| Characiformes | Serrasalmidae | *Myloplus rubripinnis* | 5 | 6 | 10 | 6 | 4 | 6 | **-** |
| Characiformes | Serrasalmidae | *Myloplus schomburgkii* | **-** | 1 | 1 | 4 | 11 | 10 | 3 |
| Characiformes | Serrasalmidae | *Prosomyleus rhomboidalis* | **-** | **-** | **-** | **-** | **-** | 3 | **-** |
| Characiformes | Hemiodontidae | *Hemiodus* sp xingu | 8 | 45 | 48 | 59 | 174 | 550 | 159 |
| Characiformes | Hemiodontidae | *Hemiodus tucupi* | **-** | 2 | 2 | 1 | **-** | **-** | **-** |
| Characiformes | Hemiodontidae | *Hemiodus unimaculatus* | 15 | 21 | 85 | 134 | 139 | 411 | 123 |
| Characiformes | Hemiodontidae | *Hemiodus vorderwinkleri* | 2 | 1 | 7 | 7 | 33 | 29 | 10 |
| Characiformes | Hemiodontidae | *Argonectes robertsi* | 3 | 7 | 63 | 27 | 87 | 107 | 11 |
| Characiformes | Hemiodontidae | *Bivibranchia fowleri* | **-** | **-** | 1 | 1 | 4 | 4 | 5 |
| Characiformes | Hemiodontidae | *Bivibranchia velox* | **-** | **-** | 1 | 51 | 34 | 174 | 49 |
| Characiformes | Anostomidae | *Schizodon trivittatus* | 22 | 8 | 3 | 1 | 2 | 3 | 1 |
| Characiformes | Anostomidae | *Hypomasticus julii* | 2 | **-** | **-** | **-** | 1 | **-** | **-** |
| Characiformes | Anostomidae | *Insperanos nattereri* | **-** | 2 | **-** | **-** | 1 | **-** | **-** |
| Characiformes | Anostomidae | *Leporinus brunneus* | **-** | **-** | **-** | **-** | **-** | 1 | **-** |
| Characiformes | Anostomidae | *Leporinus fasciatus* | 8 | 12 | 21 | 18 | 33 | 24 | 34 |
| Characiformes | Anostomidae | *Leporinus friderici* | 306 | 60 | 6 | 1 | 5 | 5 | 19 |
| Characiformes | Anostomidae | *Leporinus maculatus* | 2 | **-** | **-** | 22 | 14 | 57 | 19 |
| Characiformes | Anostomidae | *Leporinus* sp2 | **-** | **-** | **-** | **-** | **-** | **-** | 1 |
| Characiformes | Anostomidae | *Leporinus tigrinus* | 2 | **-** | **-** | 1 | 1 | 2 | **-** |
| Characiformes | Chilodontidae | *Caenotropus labyrinthicus* | 1 | 21 | 6 | 23 | 56 | 73 | 25 |
| Characiformes | Curimatidae | *Cyphocharax festivus* | 56 | 3 | 1 | 1 | 54 | 17 | 4 |
| Characiformes | Curimatidae | *Cyphocharax gouldingi* | 103 | 13 | **-** | 3 | **-** | 1 | **-** |
| Characiformes | Curimatidae | *Cyphocharax leucostictus* | **-** | **-** | **-** | **-** | **-** | **-** | 3 |
| Characiformes | Curimatidae | *Cyphocharax stilbolepis* | **-** | **-** | **-** | **-** | 1 | **-** | **-** |
| Characiformes | Curimatidae | *Curimata inornata* | 1 | **-** | 4 | 1 | 1 | 1 | **-** |
| Characiformes | Curimatidae | *Curimatella dorsalis* | 1 | 2 | **-** | **-** | 1 | **-** | 23 |
| Characiformes | Curimatidae | *Curimatella immaculata* | 80 | 32 | 11 | 3 | 15 | 14 | 20 |
| Characiformes | Prochilodontidae | *Prochilodus nigricans* | 26 | 16 | 17 | 24 | 38 | 25 | 19 |
| Characiformes | Prochilodontidae | *Semaprochilodus brama* | **-** | 3 | 2 | **-** | **-** | 6 | 12 |
| Characiformes | Ctenoluciidae | *Boulengerella cuvieri* | **-** | 11 | 6 | 3 | 12 | 53 | 12 |
| Characiformes | Chalceidae | *Chalceus epakros* | **-** | **-** | 2 | 1 | **-** | **-** | **-** |
| Characiformes | Triportheidae | *Agoniates halecinus* | **-** | 11 | 80 | 63 | 156 | 245 | 98 |
| Characiformes | Triportheidae | *Triportheus albus* | 3 | 16 | 11 | 24 | 34 | 107 | 38 |
| Characiformes | Triportheidae | *Triportheus auritus* | **-** | **-** | 1 | **-** | 8 | 21 | 21 |
| Characiformes | Triportheidae | *Triportheus curtus* | 123 | 30 | 11 | 1 | 13 | 1 | 1 |
| Characiformes | Triportheidae | *Triportheus rotundatus* | **-** | **-** | **-** | **-** | 5 | 4 | **-** |
| Characiformes | Bryconidae | *Brycon* aff pesu adiposa hialina | 2 | 6 | 12 | 5 | 8 | 34 | 2 |
| Characiformes | Bryconidae | *Brycon* aff pesu adiposa preta | **-** | 1 | **-** | 8 | 6 | 5 | 7 |
| Characiformes | Bryconidae | *Brycon falcatus* | **-** | 9 | 4 | 3 | 20 | 31 | 35 |
| Characiformes | Iguanodectidae | *Bryconops alburnoides* | 157 | 59 | **-** | 76 | 67 | 46 | **-** |
| Characiformes | Iguanodectidae | *Bryconops caudomaculatus* | 26 | 13 | 18 | 1 | 29 | 23 | 48 |
| Characiformes | Iguanodectidae | *Bryconops giacopinii* | **-** | 1 | **-** | **-** | 10 | 14 | 7 |
| Characiformes | Iguanodectidae | *Bryconops magoi* | **-** | **-** | **-** | **-** | **-** | 1 | **-** |
| Characiformes | Iguanodectidae | *Bryconops melanurus* | **-** | **-** | **-** | 5 | 2 | 1 | **-** |
| Characiformes | Characidae | *Acestrocephalus stigmatus* | **-** | **-** | **-** | 6 | 6 | 11 | 2 |
| Characiformes | Characidae | *Acestrorhynchus falcatus* | 11 | **-** | **-** | **-** | **-** | **-** | **-** |
| Characiformes | Characidae | *Acestrorhynchus microlepis* | 65 | 13 | **-** | 2 | 1 | 2 | 2 |
| Characiformes | Characidae | *Astyanax bimaculatus* | 844 | 29 | 0 | 0 | 0 | 0 | 0 |
| Characiformes | Characidae | *Charax gibbosus* | 38 | 11 | **-** | **-** | 2 | **-** | **-** |
| Characiformes | Characidae | *Ctenobrycon spilurus* | 686 | 352 | **-** | **-** | **-** | **-** | **-** |
| Characiformes | Characidae | *Jupiaba apenima* | **-** | **-** | **-** | 1 | **-** | **-** | **-** |
| Characiformes | Characidae | *Laemolyta fernandezi* | 1 | **-** | **-** | **-** | 1 | 2 | 1 |
| Characiformes | Characidae | *Laemolyta proxima* | 13 | 1 | **-** | **-** | **-** | **-** | **-** |
| Characiformes | Characidae | *Cynopotamus xinguano* | **-** | **-** | **-** | 3 | **-** | 3 | **-** |
| Characiformes | Characidae | *Moenkhausia celibela* | **-** | **-** | **-** | **-** | **-** | 1 | **-** |
| Characiformes | Characidae | *Moenkhausia heikoi* | **-** | **-** | **-** | 8 | 17 | 30 | 7 |
| Characiformes | Characidae | *Moenkhausia intermedia* | 105 | 128 | 5 | 2 | **-** | 32 | 18 |
| Characiformes | Characidae | *Moenkhausia mikia* | **-** | **-** | **-** | **-** | **-** | 2 | **-** |
| Characiformes | Characidae | *Moenkhausia xinguensis* | 8 | 1 | 1 | 3 | 8 | 21 | 4 |
| Characiformes | Characidae | *Poptella brevispina* | 12 | **-** | **-** | **-** | **-** | **-** | **-** |
| Characiformes | Characidae | *Poptella compressa* | 16 | 20 | **-** | 1 | **-** | **-** | **-** |
| Characiformes | Characidae | *Roeboexodon guyanensis* | **-** | **-** | **-** | 1 | **-** | **-** | **-** |
| Characiformes | Characidae | *Roeboides affinis* | 2 | 1 | 8 | 2 | **-** | **-** | **-** |
| Characiformes | Characidae | *Tetragonopterus chalceus* | 6 | **-** | **-** | **-** | **-** | 1 | **-** |
| Characiformes | Characidae | *Tetragonopterus kuluene* | 62 | 3 | 2 | **-** | **-** | **-** | **-** |
| Siluriformes | Cetopsidae | *Cetopsis coecutiens* | **-** | **-** | **-** | 1 | **-** | **-** | **-** |
| Siluriformes | Loricariidae | *Aphanotorulus emarginatus* | **-** | **-** | **-** | **-** | 1 | 4 | 4 |
| Siluriformes | Loricariidae | *Baryancistrus xanthellus* | 1 | **-** | 1 | **-** | **-** | **-** | **-** |
| Siluriformes | Loricariidae | *Hypoptopoma inexspectatum* | 5 | **-** | **-** | **-** | **-** | **-** | **-** |
| Siluriformes | Loricariidae | *Hypostomus cochliodon* | 4 | 4 | 5 | 2 | **-** | 1 | 3 |
| Siluriformes | Loricariidae | *Hypostomus plecostomus* | **-** | **-** | **-** | **-** | 2 | 2 | **-** |
| Siluriformes | Loricariidae | *Hypostomus plecostomus* | **-** | **-** | **-** | **-** | **-** | **-** | 4 |
| Siluriformes | Loricariidae | *Limatulichthys griseus* | **-** | **-** | **-** | **-** | 1 | 6 | **-** |
| Siluriformes | Loricariidae | *Loricaria birindellii* | **-** | **-** | **-** | **-** | 1 | 4 | **-** |
| Siluriformes | Loricariidae | *Peckoltia vittata* | **-** | **-** | 1 | 1 | **-** | **-** | **-** |
| Siluriformes | Loricariidae | *Pterygoplichthys pardalis* | 2 | 1 | **-** | **-** | **-** | **-** | **-** |
| Siluriformes | Loricariidae | *Pterygoplichthys xinguensis* | **-** | 1 | **-** | **-** | **-** | **-** | **-** |
| Siluriformes | Loricariidae | *Scobinancistrus aureatus* | **-** | **-** | **-** | **-** | 1 | **-** | **-** |
| Siluriformes | Auchenipteridae | *Auchenipterus nuchalis* | 0 | 12 | 4 | 1 | 11 | 48 | 48 |
| Siluriformes | Auchenipteridae | *Tocantinsia piresi* | **-** | **-** | **-** | **-** | 4 | 14 | 5 |
| Siluriformes | Auchenipteridae | *Ageneiosus inermis* | 119 | 69 | 7 | **-** | 1 | **-** | **-** |
| Siluriformes | Auchenipteridae | *Ageneiosus ucayalensis* | **-** | 3 | 8 | 57 | 35 | 140 | 45 |
| Siluriformes | Auchenipteridae | *Trachelyopterus ceratophysus* | 49 | 3 | **-** | 1 | 1 | **-** | **-** |
| Siluriformes | Doradidae | *Doras higuchii* | **-** | 6 | **-** | **-** | **-** | 5 | **-** |
| Siluriformes | Doradidae | *Leptodoras hasemani* | **-** | **-** | **-** | **-** | **-** | 2 | **-** |
| Siluriformes | Doradidae | *Megalodoras uranoscopus* | **-** | **-** | **-** | **-** | 1 | **-** | **-** |
| Siluriformes | Doradidae | *Platydoras armatulus* | **-** | **-** | **-** | **-** | **-** | 1 | **-** |
| Siluriformes | Doradidae | *Pterodoras granulosus* | **-** | **-** | **-** | **-** | 1 | **-** | **-** |
| Siluriformes | Doradidae | *Rhinodoras boehlkei* | **-** | **-** | **-** | **-** | 1 | **-** | **-** |
| Siluriformes | Doradidae | *Hassar gabiru* | 4 | 29 | 25 | 10 | 4 | 3 | 1 |
| Siluriformes | Pimelodidae | *Phractocephalus hemioliopterus* | **-** | **-** | 1 | **-** | 2 | **-** | 1 |
| Siluriformes | Pimelodidae | *Pimelodus blochii* | 1 | 2 | 1 | **-** | 2 | 2 | **-** |
| Siluriformes | Pimelodidae | *Pimelodus ornatus* | 1 | 2 | 3 | 4 | 1 | 3 | 4 |
| Siluriformes | Pimelodidae | *Pinirampus pirinampu* | **-** | 2 | 1 | 9 | 10 | 6 | 5 |
| Siluriformes | Pimelodidae | *Sorubim lima* | **-** | **-** | 1 | **-** | **-** | **-** | **-** |
| Siluriformes | Pimelodidae | *Sorubim trigonocephalus* | **-** | **-** | 2 | **-** | **-** | **-** | **-** |
| Cichliformes | Cichlidae | *Aequidens tetramerus* | 8 | **-** | **-** | **-** | **-** | **-** | **-** |
| Cichliformes | Cichlidae | *Caquetaia spectabilis* | **-** | **-** | **-** | **-** | 1 | 1 | 3 |
| Cichliformes | Cichlidae | *Cichla melaniae* | 2 | 3 | 1 | 5 | 1 | 8 | 30 |
| Cichliformes | Cichlidae | *Cichla pinima* | 5 | **-** | **-** | **-** | **-** | **-** | **-** |
| Cichliformes | Cichlidae | *Crenicichla lugubris* | **-** | **-** | **-** | **-** | **-** | 1 | 2 |
| Cichliformes | Cichlidae | *Crenicichla macrophthalma* | 3 | **-** | **-** | **-** | **-** | **-** | **-** |
| Cichliformes | Cichlidae | *Crenicichla saxatilis* | 1 | **-** | **-** | **-** | **-** | **-** | **-** |
| Cichliformes | Cichlidae | *Geophagus altifrons* | **-** | 1 | 26 | 70 | 94 | 100 | 91 |
| Cichliformes | Cichlidae | *Geophagus argyrostictus* | **-** | **-** | 3 | 24 | 23 | 43 | 15 |
| Cichliformes | Cichlidae | *Satanoperca jurupari* | 3 | 8 | 5 | **-** | **-** | **-** | **-** |
| Cichliformes | Cichlidae | *Satanoperca* sp | **-** | **-** | **-** | **-** | **-** | 1 | **-** |
| Eupercaria/misc | Sciaenidae | *Pachyurus junki* | **-** | 17 | 5 | 30 | 39 | 24 | 4 |
| Eupercaria/misc | Sciaenidae | *Plagioscion squamosissimus* | 88 | 52 | 78 | 102 | 91 | 82 | 43 |

**Table S2.** Scores from Principal Coordinates Analyses (PCoAs) and Principal Components Analyses (PCAs) created to reduce the dimensionality of traits associated with defense, metabolism, feeding (trophic), habitat use (habitat) and life history. Abbreviations used here are described in Table S4, S5 and S6. Percentage of explanation of each axis is presented in parentheses.

| **DEFENSE** | | **METABOLISM** | | **TROPHIC** | | | **HABITAT** | | | **LIFE HISTORY** | | |
| --- | --- | --- | --- | --- | --- | --- | --- | --- | --- | --- | --- | --- |
| **Trait** | **PCoA1 (46%)** | **Trait** | **PCoA1 (57%)** | **Trait** | **PCoA1 (33%)** | **PCoA2 (28%)** | **Trait** | **PCoA1 (41%)** | **PCoA2 (22%)** | **Trait** | **PCA1 (47%)** | **PCA2 (26%)** |
| SPI 0 | **-1.66** | AC_L 1 | 0.82 | REL_GUT_L | **-0.62** | **0.38** | REL_HEAD_L | -0.09 | -0.24 | AGE_MAX | **1.82** | -0.22 |
| SPI 1 | 0.03 | AC_L 2 | 0.76 | REL_GAPE | 0.01 | 0.01 | REL_HEAD_D | **0.40** | -0.22 | FECUNDITY | 0.74 | **-1.54** |
| SPI 2 | 0.68 | AC_L 3 | 0.94 | G_RAKER 0 | -0.08 | 0.04 | REL_BOD_D | **-0.56** | -0.37 | GROWTH_COEFF | **-1.78** | 0.23 |
| SPI 3 | -0.18 | **AC_L 4** | **-2.52** | G_RAKER 1 | 0.13 | 0.03 | REL_BOD_W | 0.10 | -0.29 | AGE_MATURITY | **1.90** | -0.14 |
| SPI 4 | **1.12** | HYP_TOL 1 | 0.04 | G_RAKER 2 | 0.00 | 0.00 | REL_PED_L | 0.22 | -0.38 | NAT_MORTALITY | **-1.88** | 0.33 |
| ARM 0 | **-1.69** | **HYP_TOL 2** | **2.91** | G_RAKER 3 | -0.06 | -0.07 | REL_PED_D | 0.33 | -0.45 | OFFSPRING_SIZE | 1.12 | 1.17 |
| ARM 1 | 0.83 | **HYP_TOL 3** | **-2.94** | MOUT_ P | **-3.13** | **2.93** | REL_PED_W | -0.02 | -0.35 | SPAWNING_TYPE1 | -0.44 | **-1.43** |
| ARM 2 | 0.07 |  |  | REL_EYE_POS | -0.01 | 0.00 | REL_DORS_L | -0.22 | **-0.86** | SPAWNING_TYPE2 | 0.25 | 0.83 |
| ARM 3 | 0.79 |  |  | REL_EYE_DEP | 0.01 | 0.00 | REL_DORS_H | -0.11 | -0.50 | SPAWNING_TYPE3 | 0.46 | **1.48** |
| AGRES 0 | **1.59** |  |  | REL_SNT_L | -0.02 | 0.02 | REL_ANAL_L | -0.15 | **0.83** |  |  |  |
| AGRES 1 | **-1.28** |  |  | REL_SNT_PR_L | -0.02 | 0.02 | REL_ANAL_D | -0.12 | -0.32 |  |  |  |
| AGRES 2 | -0.31 |  |  |  |  |  | REL_CAUD_D | -0.21 | -0.55 |  |  |  |
| SPD 0 | **1.49** |  |  |  |  |  | REL_CAUD_L | -0.08 | -0.58 |  |  |  |
| SPD 1 | -0.15 |  |  |  |  |  | REL_PEC_L | -0.02 | -0.48 |  |  |  |
| SPD 2 | -1.31 |  |  |  |  |  | REL_PELV_L | -0.03 | -0.44 |  |  |  |
| SPD 3 | -0.02 |  |  |  |  |  | PEC_POS | -0.13 | -0.06 |  |  |  |
|  |  |  |  |  |  |  | DORS_POS | -0.34 | -0.44 |  |  |  |
|  |  |  |  |  |  |  | DACT 1 | **-2.14** | **0.85** |  |  |  |
|  |  |  |  |  |  |  | DACT 2 | 0.00 | 0.00 |  |  |  |
|  |  |  |  |  |  |  | DACT 3 | **2.14** | **-0.85** |  |  |  |

**Table S3.** Physicochemical characteristics of the water measured along the intermediate reservoir (RI) in the 7 years of monitoring since its creation in 2016. The water parameter measured in the intermediate reservoir (conductivity, pH, turbidity, chlorA, dissolved oxygen and transparency) were not necessarily collected at the same collection points and therefore were not correlated directly to fish data. A Redundancy Analysis exploring the variation of these parameters across years is shown in Fig. S4. Water parameters were measured and shared by Norte Energia.

| **Year** | **Mean_Cond (min/max)** | **Mean_pH (min/max)** | **Mean_Turb (min/max)** | **Mean_ClorA (min/max)** | **Mean_DO (min/max)** |
| --- | --- | --- | --- | --- | --- |
| **2016** | 0.04 (0.02/0.12) | 6.25 (4.00/6.99) | 9.33 (1.00/74.80) | 8.42 (2.39/20.88) | 5.91 (0.87/7.91) |
| **2017** | 0.03 (0.01/0.22) | 6.49 (6.01/7.11) | 11.33 (0.70/105.00) | 7.46 (0.24/65.52) | 4.66 (0.27/7.94) |
| **2018** | 0.02 (0.01/0.04) | 7.26 (6.08/8.06) | 6.80 (2.70/27.00) | 7.17 (1.49/18.29) | 4.62 (1.55/8.73) |
| **2019** | 0.02 (0.02/0.05) | 6.80 (5.95/7.88) | 10.47 (1.80/81.80) | 7.93 (1.44/29.81) | 5.00 (2.67/7.33) |
| **2020** | 0.02 (0.01/0.06) | 7.05 (5.51/8.91) | 6.46 (1.31/25.40) | 6.75 (2.50/13.93) | 5.47 (1.65/8.36) |
| **2021** | 0.02 (0.02/0.04) | 7.20 (6.45/7.89) | 6.79 (0.43/120.00) | 5.87 (2.31/14.75) | 5.03 (1.38/8.16) |
| **2022** | 0.02 (0.02/0.05) | 6.91 (6.14/7.89) | 6.09 (0.33/58.00) | 4.76 (1.00/18.36) | 5.81 (2.67/7.88) |
| **Total** | **0.02 (0.01/0.22)** | **6.90 (4.00/8.91)** | **8.09 (0.33/120.00)** | **7.01 (0.24/65.52)** | **5.16 (0.27/8.73)** |

**Table S4.** Description of the 29 morphological characteristics measured. Used in the calculations of functional traits corresponding to the trophic axis and habitat use, the descriptions are presented in Table S5.

| **Morphological measurement** | **Abbreviation** | **Definition** |
| --- | --- | --- |
| Standard length | SL | Standard length (mm) |
| Head length | HEAD_L | Distance from the tip of the upper jaw to the most caudal extension of the operculum |
| Head depth | HEAD_D | Vertical distance from dorsum to ventrum passing through the pupil |
| Mouth gape | GAPE | Vertical distance measured inside the fully open mouth at tallest point |
| Mouth position | MOUTH_P | Angle between an imaginary line connecting the ends of the open mouth and an imaginary line between the center of the pupil and the last vertebra |
| Jaw length | JL | Length from snout to the corner of the mouth |
| Eye position | EYE_POS | Vertical distance from the center of pupil to ventrum |
| Eye diameter | EYE_D | Horizontal distance from eye margin to eye margin |
| Snout length | SNT_L | Distance from the pupil of the eye to tip of the upper jaw with mouth shut. |
| Snout protusion | SNT_PR | Distance from the back of the eye to the tip of the upper jaw with the mouth fully open and extended. |
| Body depth | BOD_D | Maximum vertical distance from dorsum to ventrum |
| Body width | BOD_W | Maximum horizontal distance from side to side |
| Caudal peduncle length | PED_L | Distance from the posterior proximal margin of anal fin to the caudal margin of the ultimate vertebra |
| Caudal peduncle depth | PED_D | Minimum vertical distance from dorsum to ventrum of the caudal peduncle |
| Caudal peduncle width | PED_W | Width of the caudal peduncle in horizontal plane at midlength. |
| Dorsal fin length | DORS_L | Distance from anterior proximal margin to posterior proximal margin of the dorsal fin. |
| Dorsal fin height | DORS_HT | Maximum distance from proximal to distal margin of the dorsal fin (excluding filaments) |
| Dorsal fin position | DORS_POS | Distance from tip of the snout to anterior proximal margin of dorsal fin |
| Anal fin length | ANAL_L | Distance from anterior proximal margin to posterior proximal margin of the anal fin |
| Anal fin height | ANAL_HT | Maximum distance from proximal to distal margin of the anal fin |
| Caudal fin depth | CAUD_D | Maximum vertical distance across the fully spread caudal fin. |
| Caudal fin length | CAUD_L | Maximum distance from proximal to distal margin of the caudal fin (excluding filaments) |
| Pectoral fin length | PEC_L | Distância máxima da margem proximal para a margem distal da nadadeira peitoral |
| Pectoral fin position | PEC_FIN_POS | Horizontal distance from the upper insertion of the pectoral fin to the lower part of the body (tip of the caudal fin) |
| Pelvic fin length | PELV_L | Maximum distance from proximal to distal margin of the pelvic fin |
| Pelvic fin position | PELV_P | Distance from tip of snout to anterior proximal margin of pelvic fin |
| Gut length | GUT_L | Length of the gut from the beginning of the esophagus to anus (fully extended without stretching) |
| Gill raker | G_RAKER | 0 = absent, 1 = short, blunt, or toothlike, 2 = intermediate or long and sparse, 3 = long and comb-like |

**Table S5.** Functional traits related to habitat use and feeding (trophic). Calculated from the morphological measures described in Table S4. An ecological description is presented for each of the traits.

| **Niche axes** | **Trait** | **Abbreviation** | **Equation** | **Ecological interpretation** | **References** |
| --- | --- | --- | --- | --- | --- |
| Habitat use | Position of dorsal fin | DORS_POS | DORS_POS / SL | Related to stability in swimming stability and part of the power in propulsion. | Standen & Lauder, 2007; Lauder & Madden, 2007; Borazjani, 2013 |
| Habitat use | Position of pectoral fin | PEC_POS | PEC_FIN_POS/SL | Directly related to swimming ability and benthic habits, braking function, position maintenance, etc. | Dumay et al. 2004 |
| Habitat use | Relative body depth | REL_BOD_D | BOD_D/SL | It is directly proportional to maneuverability and inversely to speed, a large body depth minimizes recoil energy losses. | Blake, 2004 |
| Habitat use | Relative body width | REL_BOD_W | BOD_W/SL | Assesses the influence of body shape on swimming ability. | Gatz 1979a; 1979b; Winemiller 1991 |
| Habitat use | Relative depth of anal fin | REL_ANAL_D | ANAL_HT/SL | Related to stability in swimming stability and part of the power in propulsion. | Standen & Lauder, 2007; Lauder & Madden, 2007; Borazjani, 2013 |
| Habitat use | Relative depth of caudal fin | REL_CAUD_D | CAUD_D / SL | Related to maneuverability and propulsion. | Blake, 2004 |
| Habitat use | Relative depth of dorsal fin | REL_DORS_H | DORS_HT/SL | Related to stability in swimming stability and part of the power in propulsion. | Standen & Lauder, 2007; Lauder & Madden, 2007; Borazjani, 2013 |
| Habitat use | Relative depth of the caudal peduncle | REL_PED_D | PED_D/BOD_D | High values ​​indicate great maneuver potential. | Blake, 2004 |
| Habitat use | Relative head depth | REL_HEAD_D | HEAD_D/BOD_D | Related to the position of the fish in the water column, more depressed (shallower) heads tend to have benthopelagic habits. | Gatz 1979a; 1979b; Winemiller 1991 |
| Habitat use | Relative length of anal fin | REL_ANAL_L | ANAL_L/SL | Related to stability in swimming stability and part of the power in propulsion. | Standen & Lauder, 2007; Lauder & Madden, 2007; Borazjani, 2013 |
| Habitat use | Relative length of caudal fin | REL_CAUD_L | CAUD_L/SL | Related to maneuverability and propulsion. | Blake, 2004 |
| Habitat use | Relative length of dorsal fin | REL_DORS_L | DORS_L/SL | Related to stability in swimming and part of the power in propulsion, since most of the locomotor force produced by the dorsal and anal fins is directed laterally to each side of the body. Consequently, they can generate almost as much total force as the caudal fin. | Standen & Lauder, 2007; Lauder & Madden, 2007; Borazjani, 2013 |
| Habitat use | Relative length of pectoral fin | REL_PEC_L | PEC_L/ SL | Related to maneuverability (ability to make turns), propulsion and forage, since the pectoral fins contribute to the positioning of the fish in the face of prey attack. | Lauder, Madden, Mittal, et al., 2006; Lauder & Madden, 2007; Teixeira & Bennemann 2007 |
| Habitat use | Relative length of pelvic fin | REL_PELV_L | PELV_L/SL | Related to maneuverability and control in posture.. | Webb et al., 1996; Schakmann & Korsmeyer, 2023 |
| Habitat use | Relative length of the caudal peduncle | REL_PED_L | PED_L/SL | Related to swimming ability, especially propulsive ability, maximizing thrust and minimizing drag, where a narrow caudal peduncle allows for large amplitude body movements. | Blake, 2004 |
| Habitat use | Relative width of the caudal peduncle | REL_PED_W | PED_W/BOD_W | Inversely proportional to the amplitude of swimming movements, being high for less active fish. | Blake, 2004 |
| Trophic | Relative eye depth | REL_EYE_DEP | EYE_D/HEAD_D | Eye size indicates visual acuity (i.e., the ability to resolve spatial detail), so the larger the eye size, the greater the ability to differentiate spatial details including potential prey. | Boyle & Horn, 2006; Corral-López et al., 2017; Caves et al., 2017 |
| Trophic | Relative gut length | REL_GUT_L | GUT_L/SL | Directly related to herbivory/detritivory, gut length is expected to be longer in herbivores and detritivores, and shorter in carnivores. | Hugueny & Pouilly 1999, Pouilly et al. 2003 |
| Trophic | Relative head length | REL_HEAD_L | HEAD_L/SL | Directly proportional to the ability to acquire large prey, since fish with a larger head and mouth cavity are able to consume large prey. | Gatz 1979a; 1979b; Pouilly et al. 2003; Carroll, 2004 |
| Trophic | Relative mouth gape | REL_GAPE | GAPE/BOD_D | High values ​​are related to the ability to obtain large prey and may indicate piscivorous habit | Gatz 1979a, 1979b, Pouilly et al. 2003; Carroll, 2004 |
| Trophic | Relative snout length | REL_SNT_L | SNT_L/HEAD_L | Related to prey capture ability, linked to protrusion and jaw muscle size, and pharyngeal trituration, as it contributes to the speed of attack during feeding or increases the fluid velocity around the prey. | Holzman et al., 2008; Arbour & López-Fernández, 2014 |
| Trophic | Relative snout protusion | REL_SNT_PR_L | SNT_PR/HEAD_L | Related to prey capture ability, linked to protrusion and jaw muscle size, and pharyngeal trituration, as it contributes to the speed of attack during feeding or increases the fluid velocity around the prey. | Holzman et al., 2008; Arbour & López-Fernández, 2014 |
| Trophic | Vertical eye position | REL_EYE_POS | EYE_POS/HEAD_D | Directly related to the foraging position preference of the species in the water column, nektonic fish are expected to have more eyes, while benthic fish eyes in the dorsal-most area of the head. | Gatz 1979a; 1979b; Winemiller 1991; Hugueny & Pouilly 1999 |

**References in Table S5**

Arbour, J. H., and H. López-Fernández. 2014. “Adaptive Landscape and Functional Diversity of Neotropical Cichlids: Implications for the Ecology and Evolution of Cichlinae (Cichlidae; Cichliformes).” *Journal of Evolutionary Biology* **27**:2431–2442. <https://doi.org/10.1111/jeb.12486>

Borazjani, I. 2013. “The Functional Role of Caudal and Anal/dorsal Fins During the C-start of a Bluegill Sunfish.” *Journal of Experimental Biology* **216**:1658–1669. https://doi.org/10.1242/jeb.079434

Blake, R. W. 2004. “Fish Functional Design and Swimming Performance.” *Journal of Fish Biology* **65**:1193–1222. <https://doi.org/10.1111/j.0022-1112.2004.00568.x>

Boyle, K., and M. Horn. 2006. “Comparison of Feeding Guild Structure and Ecomorphology of Intertidal Fish Assemblages from Central California and Central Chile.” *Marine Ecology Progress Series* **319:**65–84. https://doi.org/10.3354/meps319065

Carroll, A. M. 2004. “Morphology Predicts Suction Feeding Performance in Centrarchid Fishes.” *Journal of Experimental Biology* **207**:3873–3881. https://doi.org/10.1242/jeb.01227

Caves, E. M., T. T. Sutton, and S. Johnsen. 2017. “Visual Acuity in Ray-finned Fishes Correlates with Eye Size and Habitat.” *The Journal of Experimental Biology* **220**:1586–1596. https://doi.org/10.1242/jeb.151183

Corral-López, A., M. Garate-Olaizola, S. D. Buechel, N. Kolm, and A. Kotrschal. 2017. “On the Role of Body Size, Brain Size, and Eye Size in Visual Acuity.” *Behavioral Ecology and Sociobiology* **71**:179. https://doi.org/10.1007/s00265-017-2408-z

Dumay, O., P.S. Tari, J.A. Tomasini, and D. Mouillot. 2004. “Functional Groups of Lagoon Fish Species in Languedoc Roussillon, Southern France.” *Journal of Fish Biology* **64**:970–983. <https://doi.org/10.1111/j.1095-8649.2004.00365.x>

Gatz, A. J. 1979a. “Community Organization in Fishes as Indicated by Morphological Features.” *Ecology,* **60:**711–718. <https://doi.org/10.2307/1936608>

Gatz, A. J. 1979b. “Ecological Morphology of Freshwater Stream Fishes.” *Tulane Studies in Zoology and Botany* **21**:91–124.

Holzman, R., S. W. Day, R. S. Mehta, and P. C. Wainwright. 2008. “Jaw Protrusion Enhances Forces Exerted on Prey by Suction Feeding Fishes.” *Journal of The Royal Society Interface* **5**:1445–1457. <https://doi.org/10.1098/rsif.2008.0159>

Hugueny, B., and M. Pouilly. 1999. “Morphological Correlates of Diet in an Assemblage of West African Freshwater Fishes.” *Journal of Fish Biology* **54**:1310–1325. <https://doi.org/10.1111/j.1095-8649.1999.tb02057.x>

Lauder, G. V., P. G. A. Madden, R. Mittal, H. Dong, and M. Bozkurttas. 2006. “Locomotion with Flexible Propulsors: I. Experimental Analysis of Pectoral Fin Swimming in Sunfish.” *Bioinspiration & Biomimetics* **1**:S25—S34. https://doi.org/10.1088/1748-3182/1/4/s04

Lauder, G. V., and P. G. A. Madden. 2007. “Fish Locomotion: Kinematics and Hydrodynamics of Flexible Foil-like Fins.” *Experiments in Fluids* **43:**641–653. https://doi.org/10.1007/s00348-007-0357-4

Pouilly, M., F. Lino, J. G. Bretenoux, and C. Rosales. 2003. “Dietary-morphological Relationships in a Fish Assemblage of the Bolivian Amazonian floodplain.” *Journal of Fish Biology* **62**:1137–1158. <https://doi.org/10.1046/j.1095-8649.2003.00108.x>

Schakmann, M., and K. E. Korsmeyer. 2023. “Fish Swimming Mode and Body Morphology Affect the Energetics of Swimming in a Wave-surge Water Flow.” *Journal of Experimental Biology* **226**:jeb244739. <https://doi.org/10.1242/jeb.244739>

Standen, E. M. and G. V. Lauder. 2007. “Hydrodynamic Function of Dorsal and Anal Fins in Brook Trout (*Salvelinus fontinalis*).” *Journal of Experimental Biology* **210**:325–339. https://doi.org/10.1242/jeb.02661

Teixeira, I., and S. T. Bennemann. 2007. “Ecomorphology Reflect the Fish Diet in a Reservoir in South Brazil.” *Biota Neotropica* **7**:67–76. <https://doi.org/10.1590/s1676-06032007000200007>

Webb, P. W., G. D. LaLiberte, and A. J. Schrank, 1996. “Does Body and Fin Form Affect the Maneuverability of Fish Traversing Vertical and Horizontal Slits?” *Environmental Biology of Fishes* **46**:7–14. https://doi.org/10.1007/bf00001692

Wikramanayake, E. D. 1991. “Ecomorphology and Biogeography of a Tropical Stream Fish Assemblage: Evolution of Assemblage Structure.” *Ecology* **71**:1756–1764. <https://doi.org/10.2307/1937583>

**Table S6.** Functional traits related to the functional niche axes: defense, life history, metabilism and along with a trait of habitat use. Obtained from literature and from the the R package FishLife.

| **Niche axes** | **Traits** | **Abbreviation** | **Definition** |
| --- | --- | --- | --- |
| Habitat use | Daily activity | DACT | 1 = diurnal, 2 = crepuscular, 3 = nocturnal |
| Defense | Aggressiveness | AGRES | 0 = no heterospecific, 1 = heterospecific chasing and biting, 2 = chasing and biting with cutting teeth causing tissue damage |
| Defense | Armor | ARM | 0 = none, 1 = massive skull, 2 = thin bony plates covering the body, 3 = thick bony plates covering the body |
| Defense | Speed | SPD | 0 = slow and usually inactive, 1 = slow and active, often with good maneuverability, 2 = fast and agile swimmer, 3 = fast and agile swimmer who can also escape by short flights |
| Defense | Spines | SPI | 0 = absent, 1 = few short and weakly serrated, 2 = few long or few short and strongly serrated, 3 = many long (dorsal, anal), 4 = long and massive |
| Life history | Age of first maturation | AGE_MATURITY | Age of first maturation obtained from the FishLife package of R |
| Life history | Fecundity | FECUNDITY | Number of eggs per female per year. Metric obtained from the FishLife package in R |
| Life history | Growth coefficient | GROWTH_COEFF | This is a parameter of the von Bertalanffy growth function, which expresses the rate (1/year) at which the asymptotic length is reached. Information obtained from the R package FishLife |
| Life history | Maximum age | AGE_MAX | Maximum population age (tmax) obtained from the FishLife R package |
| Life history | Natural mortality | NAT_MORTALITY | The instantaneous natural mortality rate (M; 1/year). This metric refers to the late juvenile and adult phases of a population and is calculated from the empirical equation of Pauly (1980). Information obtained from the FishLife R package. |
| Life history | Offspring size | OFFSPRING_SIZE | Body size of offspring. Information obtained from the FishLife package of R. |
| Life history | Reproduction type | SPAWNING_TYPE | Fishes are separated into:  1 = Nonguarders,  2 = Guarders, or  3 = Bearers. Information obtained from the R package FishLife. |
| Metabolism | Activity level | AC_L | 1 = sedentary, infrequent slow swimming, 2 = slow swimming at frequent intervals, 3 = continuous slow swimming, 4 = constant swimming with fast bursts |
| Metabolism | Hypoxia tolerance | HYP_TOL | 1 = High, 2 = Medium, 3= Low |


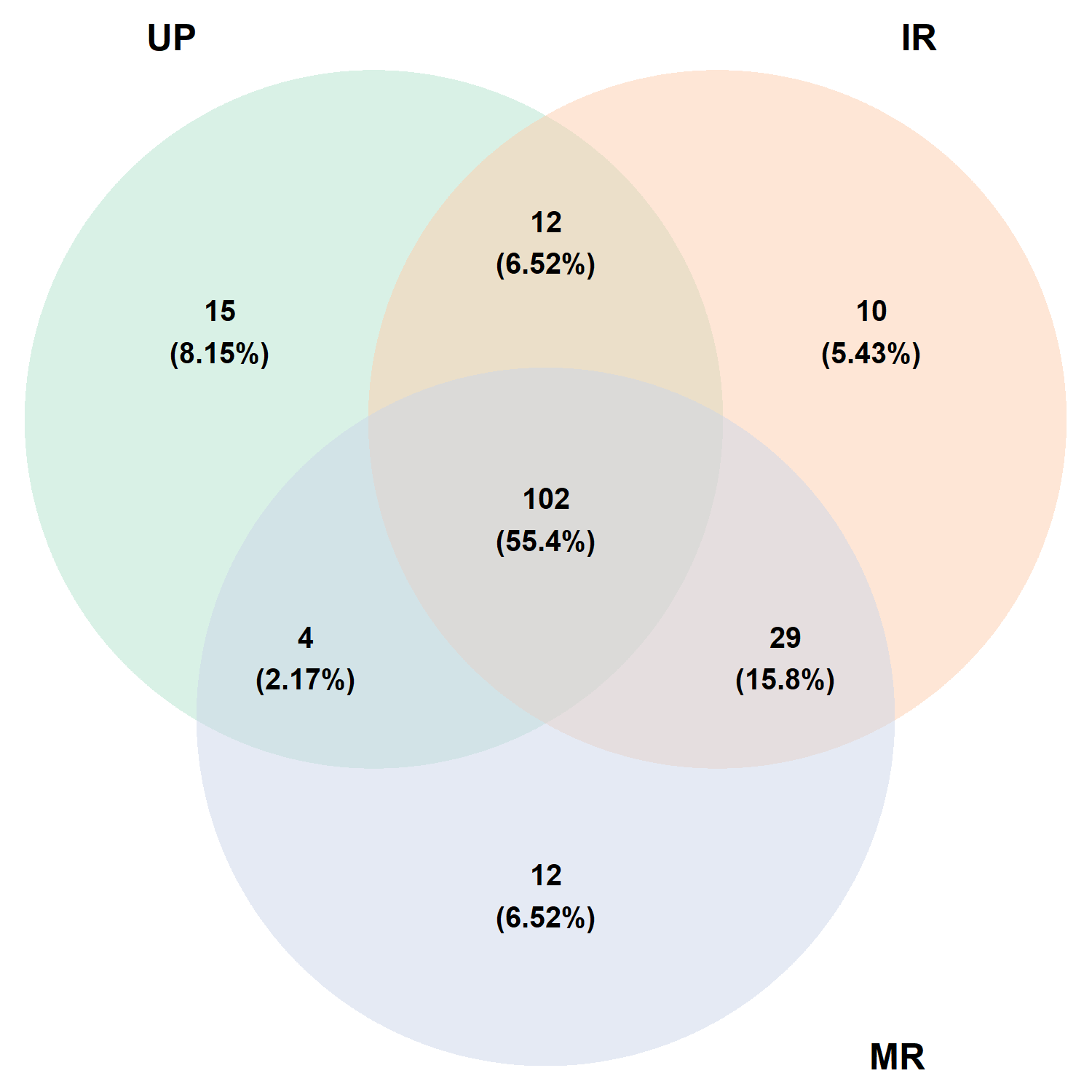


**Fig. S1.** Venn diagram showing the similarity of species composition between the intermediate reservoir (IR) and the upstream sector (UP) and main reservoir (MR).

**
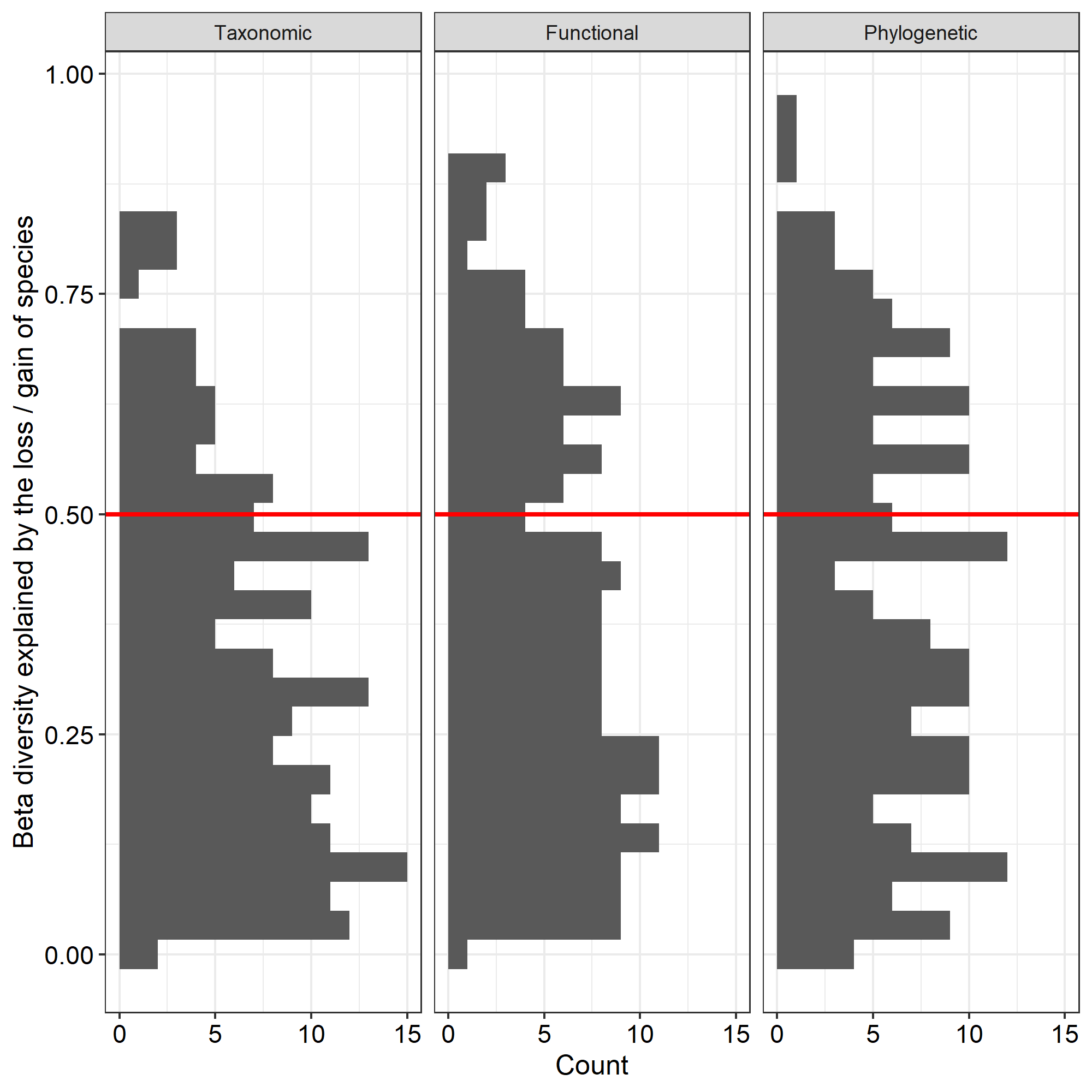
**

**Fig. S2 -** Histograms showing the distribution of beta diversity component values ​​between the river (Main Reservoir - MR and Upstream - UP) and the Intermediate Reservoir (IR) explained by species loss/gain for each biodiversity facet analyzed (i.e., taxonomic, functional, and phylogenetic diversity) between 2016 and 2022. The y-axis values ​​range from 0 (beta diversity is explained exclusively by species replacement) to 1 (beta diversity is explained exclusively by species loss or gain). Each observation represents the compositional differences (beta diversity) between a pair of sites in a given sampling year.


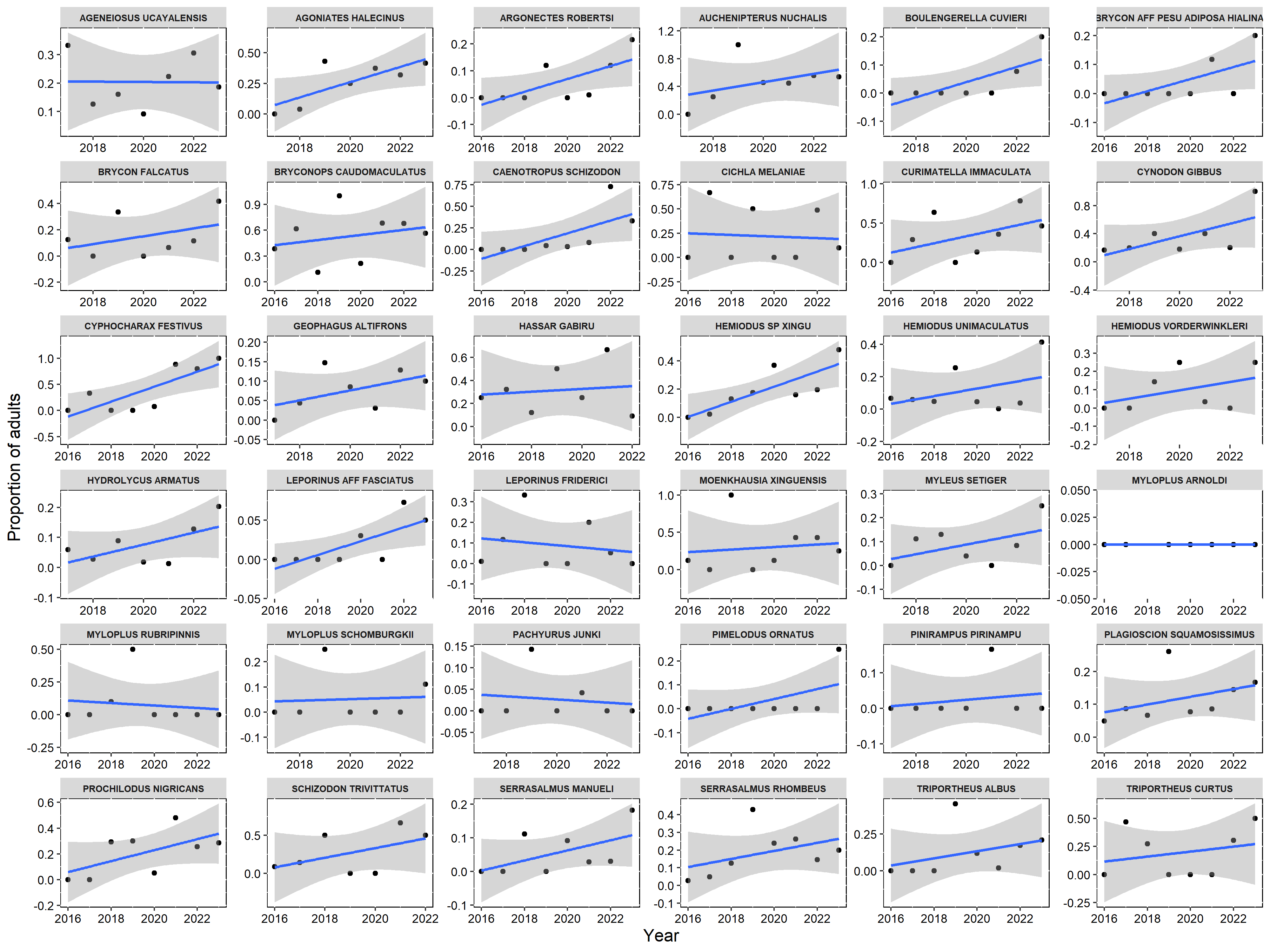


**Fig. S3**. Association between year of monitoring and proportion of adults for 36 fish species collected in the intermediate reservoir. All species analyzed here occurred in at least 7 of the 8 years of monitoring. Trend lines and their respective standard errors were generated from linear regressions.


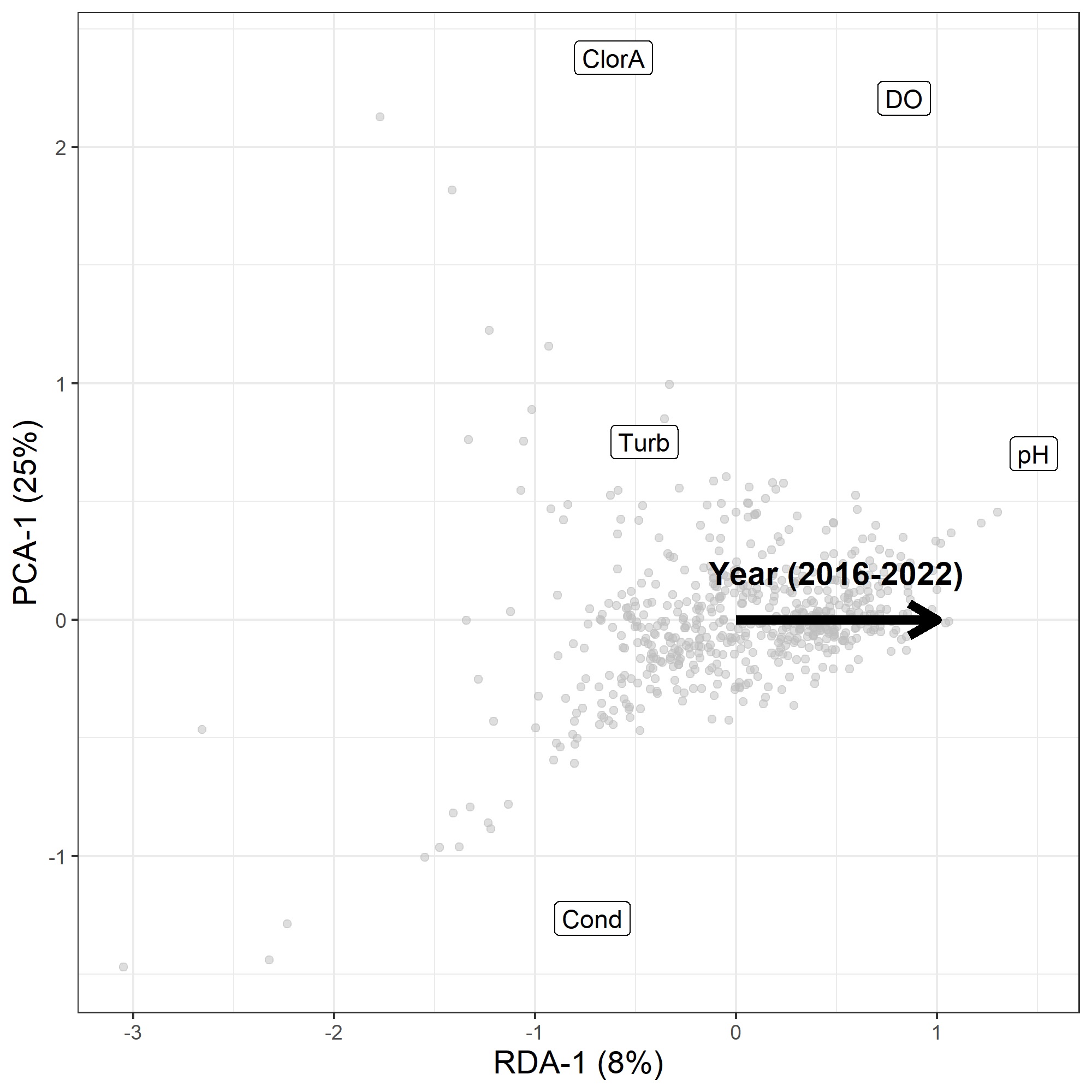


**Fig. S4**. Redundancy Analysis (RDA) of the water quality parameter variation across the years in the Intermediate Reservoir (IR). Water quality parameters were scaled before analysis. An anova-like permutation test (1000) indicates that year was a significant predictor of water parameters (F(1,556)=45.47, P<0.001). Overall, the initial years after the reservoir filling were characterized by high values of turbidity, conductivity, and chlorophyll A, and lower values of pH and dissolved oxygen (DO). Water quality parameters were provided by Norte Energia and are shown in Table S3.


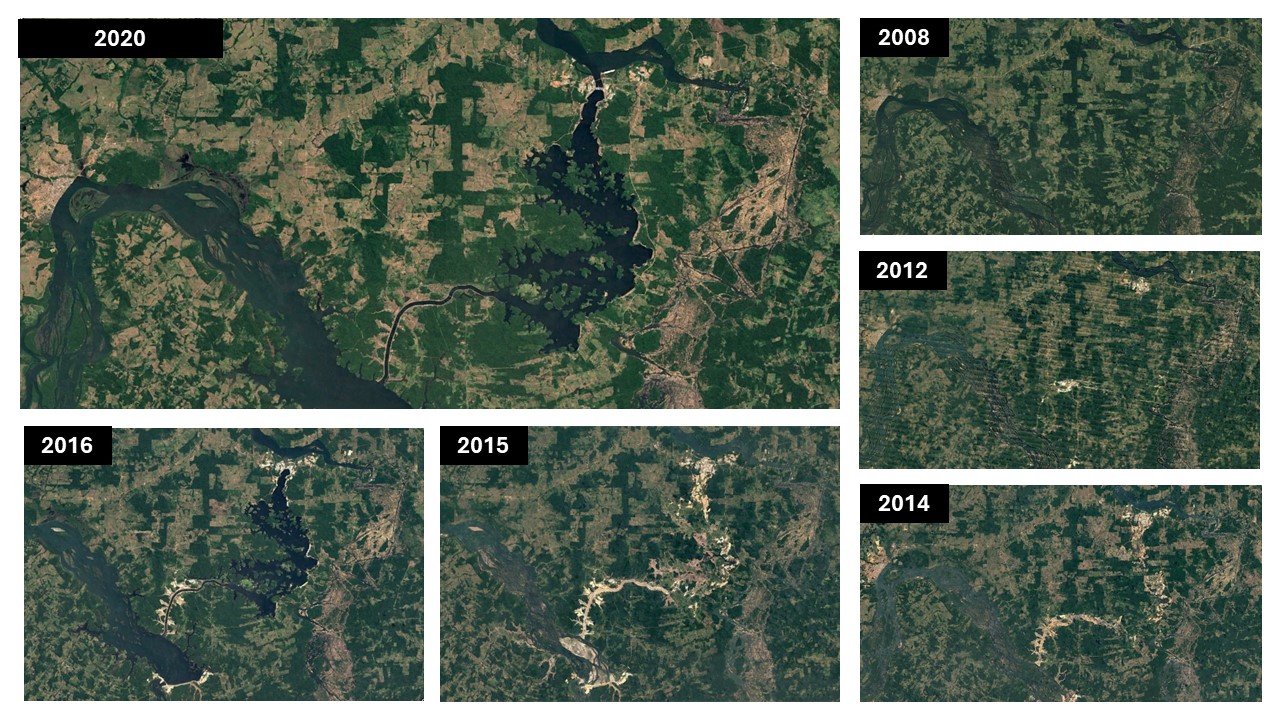


**Fig. S5.** Historical aerial images (2008 - 2020) of the construction area of the Intermediate Reservoir (IR). Construction started in 2012 and reservoir filling in 2016. Satellite images were obtained from Google Earth 7.3.6 (<https://earth.google.com/>). Panels were combined and edited into a single figure using GIMP 3.0.2 (<https://www.gimp.org/>).


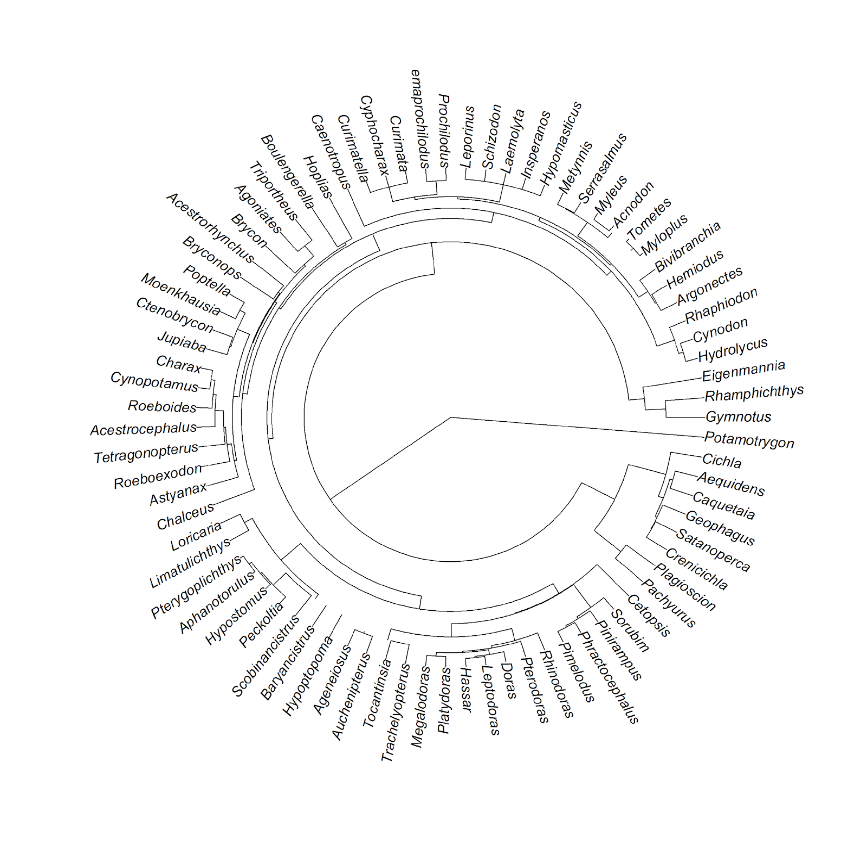


**Fig. S6.** Phylogenetic relationships among fish genera collected in the Middle Xingu River. The phylogenetic tree is based on the super tree created by Rabosky et al. (2018). Because the supertree did not include Chondrichthyes, we added a clade of freshwater stingrays (Potamotrygonidae) to the phylogeny based on their time of divergence from the ancestor of ray-finned fishes (~473 MYA; Kumar et al., 2017).

**References in Fig. S6**

Kumar, S., G. Stecher, M. Suleski, and S. B. Hedges. 2017. “TimeTree: A Resource for Timelines, Timetrees, and Divergence Times.” *Molecular Biology and Evolution* **34**: 1812–1819.<https://doi.org/10.1093/molbev/msx116>

Rabosky, D. L., J. Chang, P. O. Title, P. F. Cowman, L. Sallan, M. Friedman, K. Kaschner, C. Garilao, T. J. Near, M. Coll, and M. E. Alfaro. 2018. “An Inverse Latitudinal Gradient in Speciation Rate for Marine Fishes.” *Nature* 559:392–395.<https://doi.org/10.1038/s41586-018-0273-1>


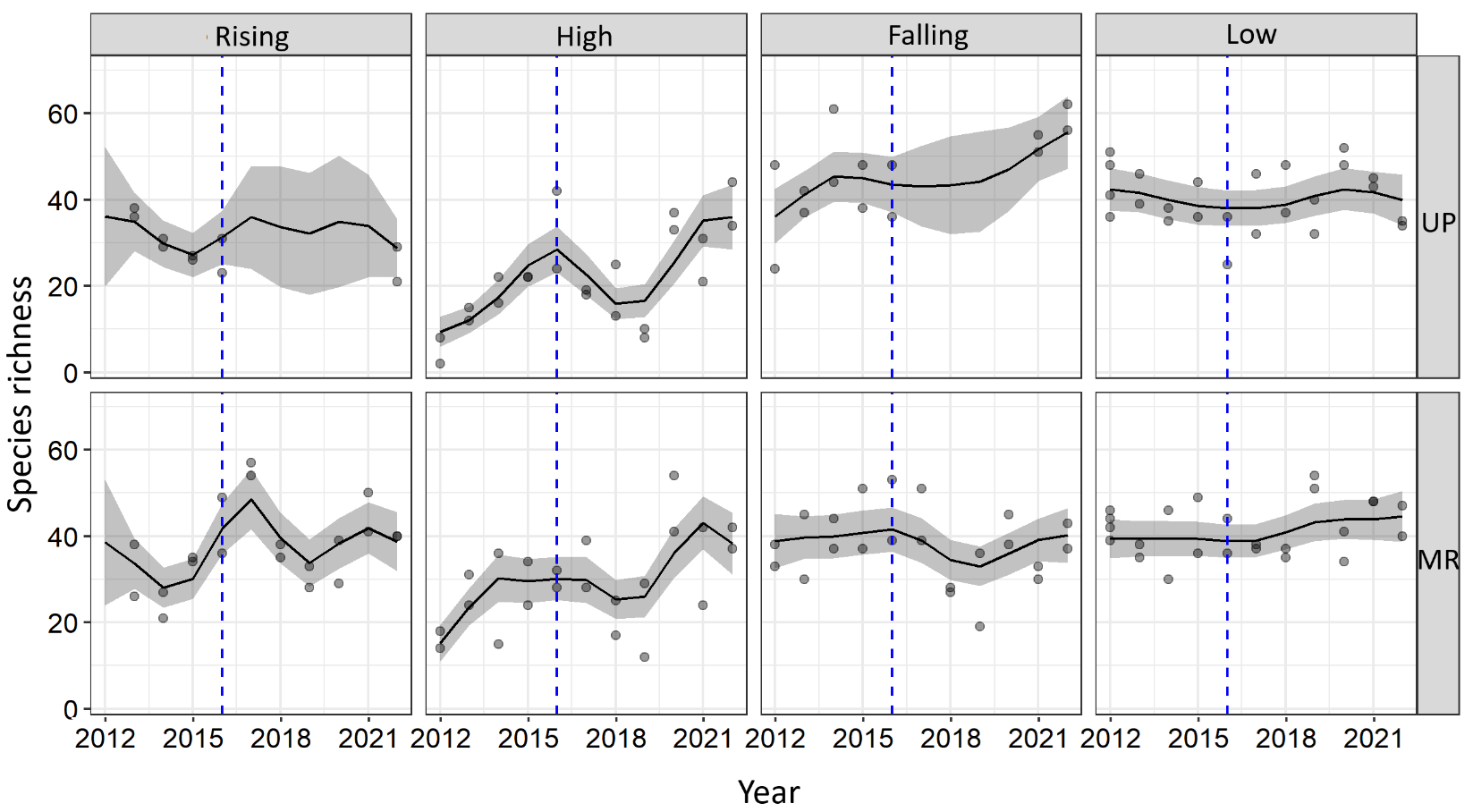


**Fig. S7.** Temporal variation in fish richness in the upstream (UP) and main reservoir (MR) sectors across hydrological periods. Trend lines and gray bars represent standard errors, which were derived from Hierarchical Generalized Additive Models (HGAM). Figure adapted from ^43^ .

**
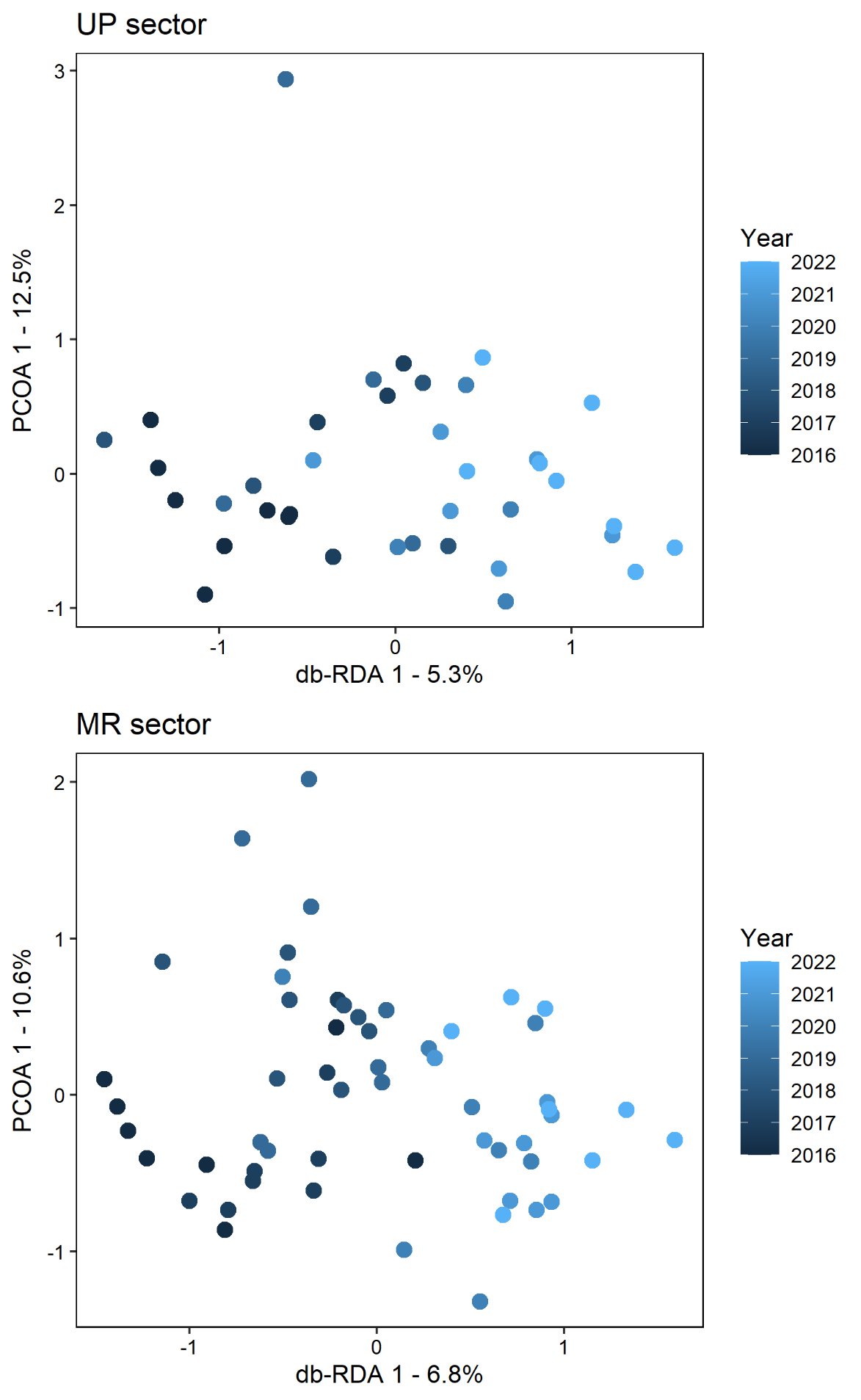
**

**Fig. S8** Distance-Based Redundance Analysis (db-RDA) of fish assemblages in the Upstream sector (UP) and Main Reservoir (MR). The fish assemblage data was restrained by sampling year and controlled by hydrological period. Numbers in parentheses represent the amount of variation explained by the ordination axes. Only axis 1 represents the variation attributed to sampling year. Figure adapted from ^43^ .
